# Diagnosis, management and prevention of loiasis: guideline of the German Society for Tropical Medicine, Travel Medicine, and Global Health (DTG)

**DOI:** 10.1007/s15010-024-02443-2

**Published:** 2025-05-21

**Authors:** Michael Ramharter, Stefan Schlabe, Marc P. Hübner, Pia Michelitsch, Florian Kurth, Sabine Bélard, Tamara Nordmann, Saskia Dede Davi

**Affiliations:** 1 Bernhard Nocht Institute for Tropical Medicine & I. Dep. of Medicine University Medical Center Hamburg-Eppendorf, Hamburg, Germany; 2 Centre de Recherche Médicales de Lambaréné, Lambaréné, Gabon; 3 German Center for Infection Research (DZIF), Partner Site Bonn-Cologne, Bonn, Germany; 4 Institute for Medical Microbiology, Immunology and Parasitology, University Hospital Bonn, Bonn, Germany; 5 German Center for Infection Research, Partner Site Hamburg-Lübeck-Borstel-Riems, Hamburg-Lübeck-Borstel-Riems, Germany; 6 Charité- Universitätsmedizin Berlin, corporate member of Freie Universität Berlin and Humboldt- Universität zu Berlin, Berlin, Germany; 7 Institute of Tropical Medicine, University of Tübingen, Tuebingen, Germany; 8 German Center for Infection Research, Partner Site Tübingen, Tübingen, Germany; 9 MVZ Labor Limbach Nord GmbH, Hanover-Lehrte, Germany

**Keywords:** Loa loa, Loiasis, Filaria, Guideline, Diagnosis, Treatment Prevention

## Abstract

Loiasis is a complex filarial infection endemic in Central Africa and parts of West Africa. *Loa loa* is transmitted by the deer fly *Chrysops dimidiata* and *C. silacea*. The clinical manifestation of the disease is highly variable ranging from asymptomatic infection, symptomatic disease, to life-threatening complications. The diagnosis of *L. loa* infection is challenging due to a significant proportion of occult infections and a lack of reliable point of care tests. While diethylcarbamazine is the gold standard for curative treatment in many non-endemic countries, its use is limited in endemic regions due to its propensity for severe adverse drug reactions that may occasionally lead to life threatening complications. Alternative treatment regimens have specific indications and limitations in the treatment of loiasis. In this guideline, issued by the German Society for Tropical Medicine, Travel Medicine, and Global Health, recommendations for the diagnosis, management, treatment, and prevention of loiasis are provided based on the currently available best evidence, and gaps in our understanding are highlighted.

## Introduction

The African Eye Worm *Loa loa* has been known to populations residing in endemic regions since time immemorial. Western medicine first encountered this paradigmatic helminth disease during the 18th century [1]. While research efforts at the end of the 19th and the beginning of the 20th century were successful in identifying the pathogen causing loiasis, its life cycle, vector and the pathognomonic clinical signs and symptoms, research efforts have virtually stalled during the second half of the past century [2]. This lack of interest in loiasis was mostly due to the fact that it has been regarded as a rather benign nuisance compared to other tropical diseases that lead to more acute life-threatening disease presentation. It was only when the ivermectin mass drug administration programs for the large scale control of onchocerciasis were severely impacted by the occurrence of serious adverse events in individuals with highly microfilaraemic loiasis, that international research efforts once more started to address loiasis [3]. 

Today, it is well established that loiasis causes important morbidity and mortality [4–6]. The treatment of this multi-faceted filarial infection is known to be complex and may be associated with potentially life-threatening adverse drug reactions in a sub-group of patients. However, the renewed research efforts have led to an improved understanding of the disease [2]. 

In parallel with the neglect in research, loiasis has also suffered from a neglect in the establishment of evidence-based recommendations for its clinical presentation, diagnosis, treatment, and prevention. To date there are no guidelines for the management of loiasis published neither by the major medical and scientific societies nor by the World Health Organization. While proposals for the management of loiasis have been published by individual experts, available summaries of treatment practices as described in authoritative textbooks or institutional recommendations seem contradictory or outdated [7–10]. 

To close this gap, the German Society for Tropical Medicine, Travel Medicine, and Global Health therefore embarked on establishing an evidence-based S1-guideline following the methodological framework of the Association of Scientific Medical Societies in Germany (AWMF) for the management of loiasis to offer guidance for health care providers dealing with patients suspected to suffer from loiasis. The original guideline is published in German language [11]. An English version is published here to allow for an international scrutinization, discussion, and the use of this guideline.

## Methods

This guideline of the German Society for Tropical Medicine, Travel Medicine, and Global Health (DTG) was commissioned by the standing Guideline Committee of the society. A sub-group of specialists for the treatment, diagnosis, prevention, management, and clinical research in the field of loiasis was established within the Guidelines Committee and further specialists were invited to join the guideline committee. The expert group included clinicians of the specialties internal medicine, infectious diseases, tropical medicine, paediatrics, parasitology as well as clinical research. All experts actively involved in the assessment of publications, discussions within the guideline group, and in the writing of the recommendations disclosed any potential conflict of interest [12]. The disclosure was summarized in a table and was judged by an independent researcher for potential implications. The guideline development followed the methodological framework of the Association of Scientific Medical Societies in Germany (AWMF) following S1-classification which constitutes a consensus-based guideline approach [13].

A systematic literature review was performed to identify all published evidence on human loiasis in scientific peer-reviewed journals without restriction of time of publication or language. The MeSH terms “*Loa loa*” and “loiasis” and the general search terms “loiasis, *Loa loa*, loa*, loiasis, loase, loaose, *Filaria loa*, *Filaria diurnal”* were used to identify relevant scientific articles. PubMed and Google Scholar were used as search engines. In addition to this systematic literature search, conference abstracts, references of literature reviews, and seminal textbooks on loiasis were included in the literature review.

In addition, a systematic evaluation of all clinical trials reporting the treatment of loiasis was performed. Recommendations were based on the available evidence and a structured discussion among the expert group was performed until consensus was reached. The German version of the guideline is published at the guideline repository of AWMF and the homepage of the German Society for Tropical Medicine, Travel Medicine, and Global Health (www.dtg.org) [11]. 

### Life cycle of *Loa loa*

**Fig. 1:**
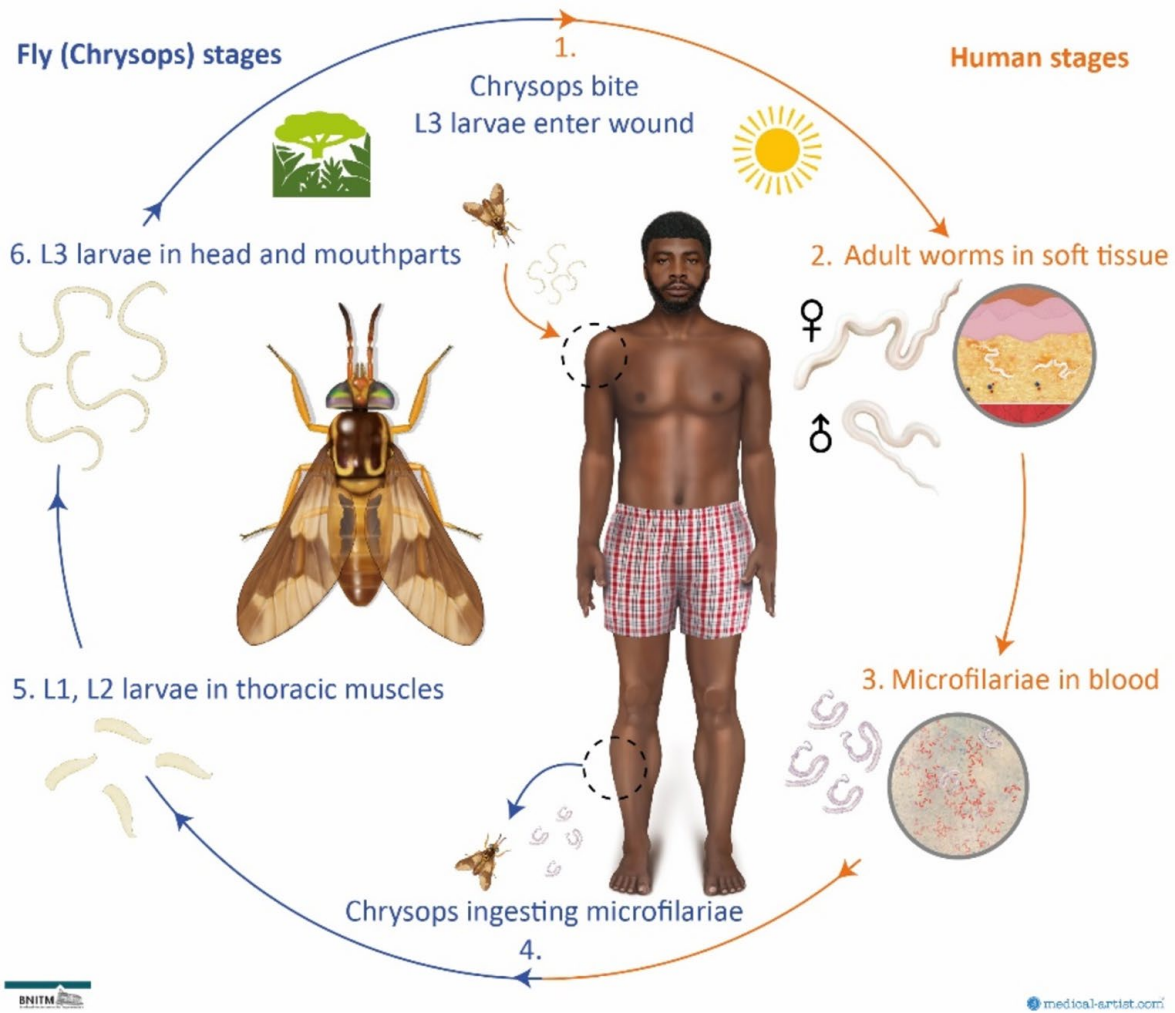
Lifecycle of *Loa loa*. Medical illustrations by Dr Joanna Butler 2024 (www.medical-artist.com). Rights to this figure are owned by medical-artist.com and the Bernhard Nocht Institute for Tropical Medicine. Permission was granted to use these graphics for this publication

Loiasis is caused by the transmission of the filarial nematode *Loa loa* via tabanid flies of the genus *Chrysops*. *C. silacea* and *C. dimidiata* are the epidemiologically relevant vectors in the endemic regions of sub-Saharan Africa [2]. These deerflies occur almost uniquely in the rainforest and adjacent savannah ecosystems of rural regions [14]. The transmission of L3 larvae takes place by a bite of the *Chrysops* fly, when infectious larvae penetrate the skin via the wound of the insect bite (Fig. 1). After migration via lymphatic vessels to the subcutaneous and fascial soft tissue, larvae develop to adult worms within months and start mating [15, 16]. Their offspring, microfilariae, reside in high numbers in peripheral blood during daytime (but may also be found in the skin and bodily fluids), where they may be taken up by the diurnally active female *Chrysops* flies during another blood meal. Ingested L1 larvae subsequently penetrate from the midgut to the fat body and thoracic muscles to develop into infectious L3 larvae [2, 17]. 

## Epidemiology of loiasis

Loiasis has been known as a clinical disease for millennia by autochthonous populations of endemic regions. Its transmission cycle and developmental stages have been elucidated in the 19th and 20th century by Western medicine [1, 18]. Since its description, the infection was characterized as a rather benign disease prevalent in rural regions of sub-Saharan Africa [3]. It was not until very recent times that epidemiological studies have demonstrated its public health importance due to its association with premature mortality and the disease burden caused by loiasis as described by attributable disability-adjusted life-years lost to disease (DALYs) [4–6]. In fact, the disease burden in Central African regions of highest transmission is comparable to that of other widely recognized Neglected Tropical Diseases such as schistosomiasis [2, 6]. 

**Fig. 2:**
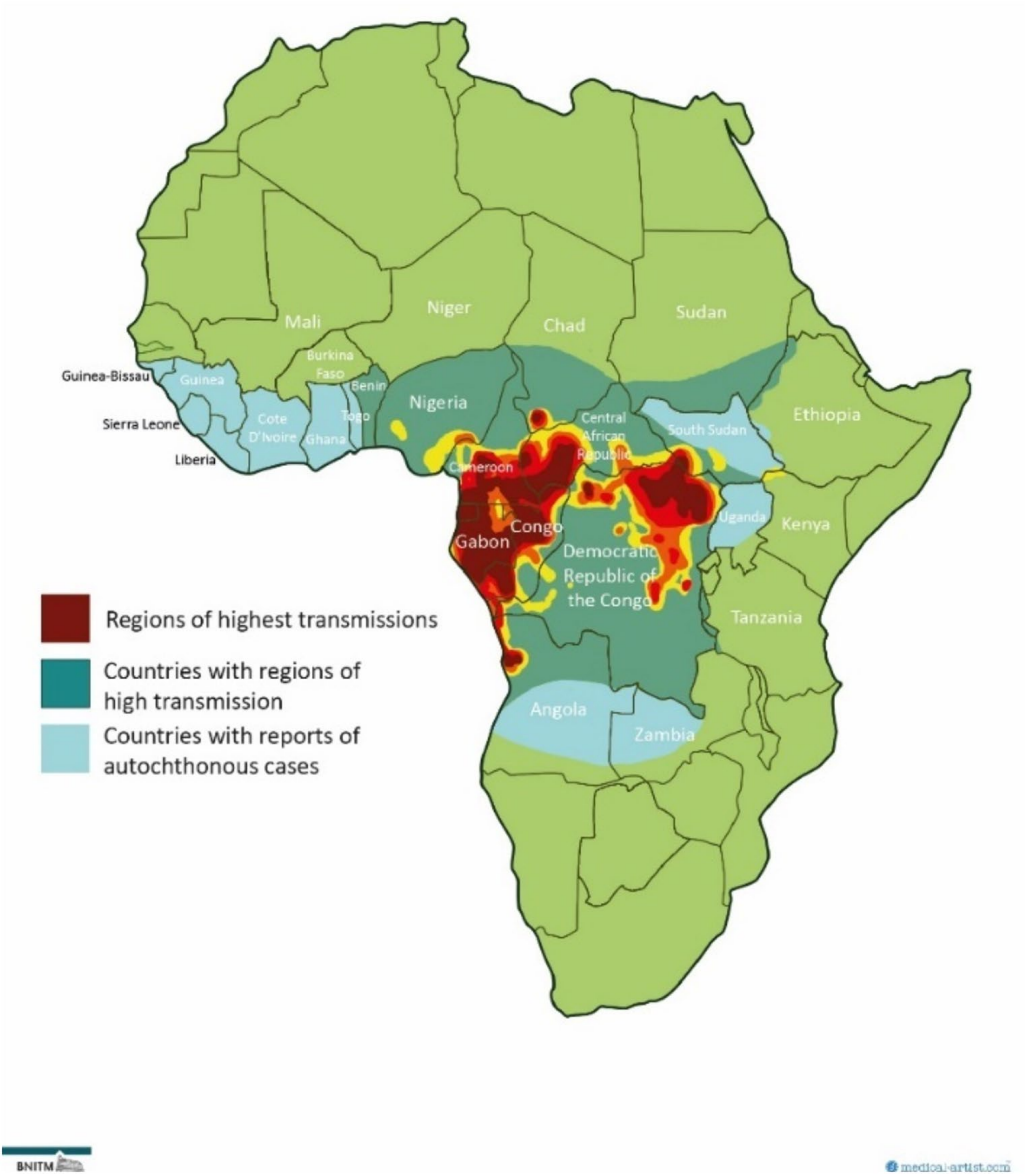
Regions with active transmission of *Loa loa.* Medical illustrations by Dr Joanna Butler 2024 (www.medical-artist.com). Rights to this figure are owned by medical-artist.com and the Bernhard Nocht Institute for Tropical Medicine. Permission was granted to use these graphics for this publication

Loiasis is endemic in the rural regions of Central Africa and parts of West Africa [2]. The endemic region is determined by the habitat of the vector *C. siliacea* and *C. dimidiata* ranging from the Republic of Benin in the west to Uganda in the east and Angola in the south (Fig. 2). High transmission has been reported in the rain forest, adjacent savanna ecosystems and their transition zones [19]. Human land change including deforestation, agriculture, or agricultural plantations of rubber or oil palm may result in considerable alterations of transmission activity [20, 21]. 

Until now, there are no representative surveys of the infection prevalence based on a combination of clinical and parasitological examinations. Therefore, data on the prevalence have to be estimated indirectly. A standardized questionnaire, recommended by WHO for the population-based assessment of the epidemiology of loiasis, was used for these surveys. This standardized survey (Rapid Assessment Procedure for Loiasis, RAPLOA) is based on the anamnestic reporting of the characteristic eye worm migration of the adult worm [22, 23]. The highest prevalence of eye worm migration in > 40% of the population were found in two hyperendemic zones and affected a population at risk of 20.5 million people. A western hyperendemic zone is located in Gabon, Equatorial Guinea, parts of Cameroon, the Central African Republic, while the eastern hyperendemic zone is located in the northeast of the Democratic Republic of Congo (DRC). Further 21.7 million people live in regions of intermediate risk (history of eye worm migration in 20–40%) [19]. Taken together, the number of infected persons in the zones of intermediate and high transmission prevalence is estimated at more than 20 million patients, 80% of whom live in Cameroon and DRC [2]. In countries west of Nigeria, occasional case reports indicate the potential of active transmission. However, the transmission prevalence is largely unknown [2]. 

There are published reports on *L. loa* infections outside the above-mentioned area of West and Central Africa. While uncharacterised regions of endemicity may not be entirely ruled out, not least due to potential of a variety of local *Chrysops* species to transmit *L. loa*, these case reports so far do not constitute unequivocal proof due to an absence of microscopical or molecular species determination [2, 24]. Therefore, a misinterpretation of an eye worm migration has to be taken into consideration until proof of the contrary (please also refer to differential diagnoses of eye worm migration below). Since *Chrysops spp.* originating from North America have been shown as potential competent vectors of *L. loa*, a future expansion of the region of active transmission cannot be ruled out [24]. 

The infection prevalence of *L. loa* varies considerably within endemic regions [6, 19]. It mainly affects economically underprivileged populations of the rural rainforest and savanna regions. The infection rate increases with age from childhood to the end of the teenage years and remains constant throughout adulthood and into older age [6, 25, 26]. The risk of infection is in general associated with a lower socioeconomic status, while a concomitant HIV-infection does not appear to be an independent risk factor [27, 28]. 

Outside of endemic regions loiasis may be observed occasionally in travellers and migrants from endemic regions. The documented number of cases with diagnosed imported loiasis is comparatively low. There are no official surveillance data from Germany as loiasis is not a notifiable disease by German law. An international case series comprising 101 patients consulted between 1986 and 2011 contained only 11 cases with country of origin or residence in Germany [29]. Similarly, the Center for Tropical Medicine in Hamburg reported diagnosis and treatment of 11 episodes of loiasis over a period of 10 years, illustrating the rarity of loiasis in Germany [30]. 

Other European countries with historically closer links to the most endemic countries in sub-Saharan Africa report higher numbers of imported loiasis. As example a report from the metropolitan region of Paris included 177 cases between 1993 and 2013 [31]. Another study in three hospitals in Marseille and Lyon included 47 cases 1998–2012 [32]. Similarly, studies from Italy (100 cases between 1993 and 2013), Belgium (150 cases between 1994 and 2012) and the United Kingdom (50 cases between 2000 and 2014) demonstrate higher case numbers in these countries [33–35]. The GeoSentinel Network, a global network for the surveillance and study of travel-related illness, registered 68 loiasis cases among 43,722 travellers in the period 1997–2004 again highlighting the overall infrequency of loiasis as travel related infection [36]. In this cohort, the majority of loiasis patients were migrants and a smaller proportion were long-term travellers with a stay of at least one month in the endemic country. Interestingly, several studies demonstrated that travellers present with more clinical symptoms compared to persons who grew up in endemic regions. This different clinical presentation is thought to be immune-mediated [37]. 

## Clinical presentation and differential diagnosis of loiasis

The presentation of clinical symptoms following an infection are closely linked with the developmental life cycle of *L. loa*. First clinical symptoms following the transmission of the infective L3 larvae may appear only after 3–6 months [2]. The prepatent period, which describes the time until microfilariae appear in peripheral blood, is usually more than one year, but may vary widely between a few months and up to 15 years [38, 39]. 

The number of microfilariae in peripheral blood increases from the timepoint of the first appearance over a few months until it reaches a relatively constant level. However, a relevant proportion of infected persons, often between 30 and 60%, do not develop microfilaraemia, and therefore are classified as cases of occult loiasis [2, 40]. Absence of microfilariae may occur in case of infection with a single nematode, several nematodes of the same sex, or host factors such as an efficient immunological control of worm reproduction or genetic predisposition [2]. The average lifespan of the adult worms in the human host is unknown, but up to 20 years are documented [41, 42]. 

Loiasis may cause a wide range of clinical symptoms with varying clinical penetrance. The clinical presentation is in general not linked to the presence of microfilariae in peripheral blood, a fact that often leads to confusions in the diagnosis and classification of loiasis [2, 40]. 

### Classification of loiasis from a parasitological and a clinical perspective

A classification of loiasis into parasitological or clinical categories (Table 1) is helpful, firstly, to characterise respective disease presentations and, secondly, to appropriately diagnose and treat loiasis.

**Table 1:** Classification of loiasis by parasitological and clinical criteria

Classification	Category	Defintion	Specifics
Parasitological classification	Occult loiasis	Assumed or confirmed infection with *L. loa* without parasitological confirmation of peripheral microfilaraemia	Confirmation of diagnosis often challenging
	Microfilaraemic loiasis	Confirmation of *L. loa* infection by parasitological detection of *L. loa* microfilariae in peripheral blood	Confirmation of diagnosis usually straightforward, treatment depends on microfilarial load
	Hypermicrofilaraemic loiasis	Detection of microfilariae in high quantity during diurnal periodicity (variable threshold for hypermicrofilaraemia reported: >8,000 mf/mL, > 20,000 mf/mL, or > 30,000 mf/mL	Confirmation of diagnosis straightforward; increased risk for treatment-induced complications, increased risk for secondary organ damage
Clinical classification	Migratory loiasis	*L. loa* infection with typical clinical signs of migrating adult worms (e.g. eye worm migration)– independent from presence/absence of microfilaraemia	Substantial morbidity due to specific and unspecific symptoms of migratory loiasis; in the absence of microfilaraemia often challenging confirmation of the diagnosis
	Non-migratory loiasis	*L. loa* infection without symptoms of migrating adult worm (e.g. dermal or eye worm migration, Calabar swelling)	Often silent disease without significant subjective symptoms. Association with organ complications and mortality has been described

The classical categorization into occult and microfilaraemic loiasis is based on the parasitological detection of microfilariae in peripheral blood. “Occult loiasis” therefore refers to an infection with *L. loa* as evidenced by characteristic or even pathognomonic clinical signs including eye worm migration or Calabar swelling (and potential other supportive indirect signs of loiasis as described below) in the absence of microfilariae in parasitological examination of daytime blood samples [2]. On the contrary, “microfilaraemic loiasis” is defined by the presence of *L. loa* microfilariae irrespective of the presence or absence of clinical symptoms. Microfilaraemia may vary remarkably between a few larvae to more than 100,000 microfilariae per mL of blood. High microfilaraemia is referred to as “hypermicrofilaraemia” for which varying thresholds proposed in literature most commonly are between 8,000 and 20,000 mf/mL [2]. Interestingly, on a population level, occult loiasis is the predominant form of infection in endemic regions and even more so in returning travellers [36, 37, 43, 44]. 

Besides the parasitological classification of loiasis in an occult and a microfilaraemic form, a categorization from a clinical perspective is useful. “Migratory loiasis” refers to patients with clinical signs of migrating adult worms (i.e. eye worm migration, Calabar swelling, or dermal adult worm migration) irrespective of the presence or absence of microfilaraemia [40]. On the contrary, “non-migratory loiasis” is defined as the presence of microfilaraemia in the absence of signs of adult worm migration (see above). This clinical categorization is helpful to understand the fact that patients with migratory loiasis in general develop a significant subjective disease burden by the classical and unspecific symptoms (asthenia, myalgia, arthralgia, pruritus) of loiasis. At the same time, these patients are often misdiagnosed as “uninfected” in case of absence of microfilaraemia. Contrarily, patients with a non-migratory form of loiasis do in general not complain of subjective symptoms more often than uninfected individuals and will therefore be less likely to actively seek medical consultation [40]. These asymptomatic patients, however, face the highest risk of life-threatening complications of loiasis such as secondary organ failure and the highest loiasis-associated mortality [4, 5]. 

The presence of microfilaraemia seems to be partly determined by immunological tolerance [45, 46]. From a pathophysiological view, it is therefore plausible that clinical symptoms may be more pronounced in occult forms in which an active immunological response controls microfilaraemia, compared to microfilaraemic, non-migratory loiasis with little immune activation.

Compared to patients, who lived in endemic regions since birth, travellers from non-endemic countries often present with higher eosinophilia, higher antibody titres and more pronounced clinical symptoms [37, 47]. To date, it remains unclear whether the lower immunological responsiveness to *L. loa* infection in residents of endemic regions is caused by early exposure in lifetime (e.g. *in utero* during pregnancy), by immunological, or genetic factors. While historically it was thought that most infected individuals in endemic regions remain asymptomatic, this concept is today refuted, as loiasis causes considerable morbidity and is one of the three most common reasons for medical consultation in some endemic regions [6, 48]. 

### Clinical features of loiasis

**Fig. 3:**
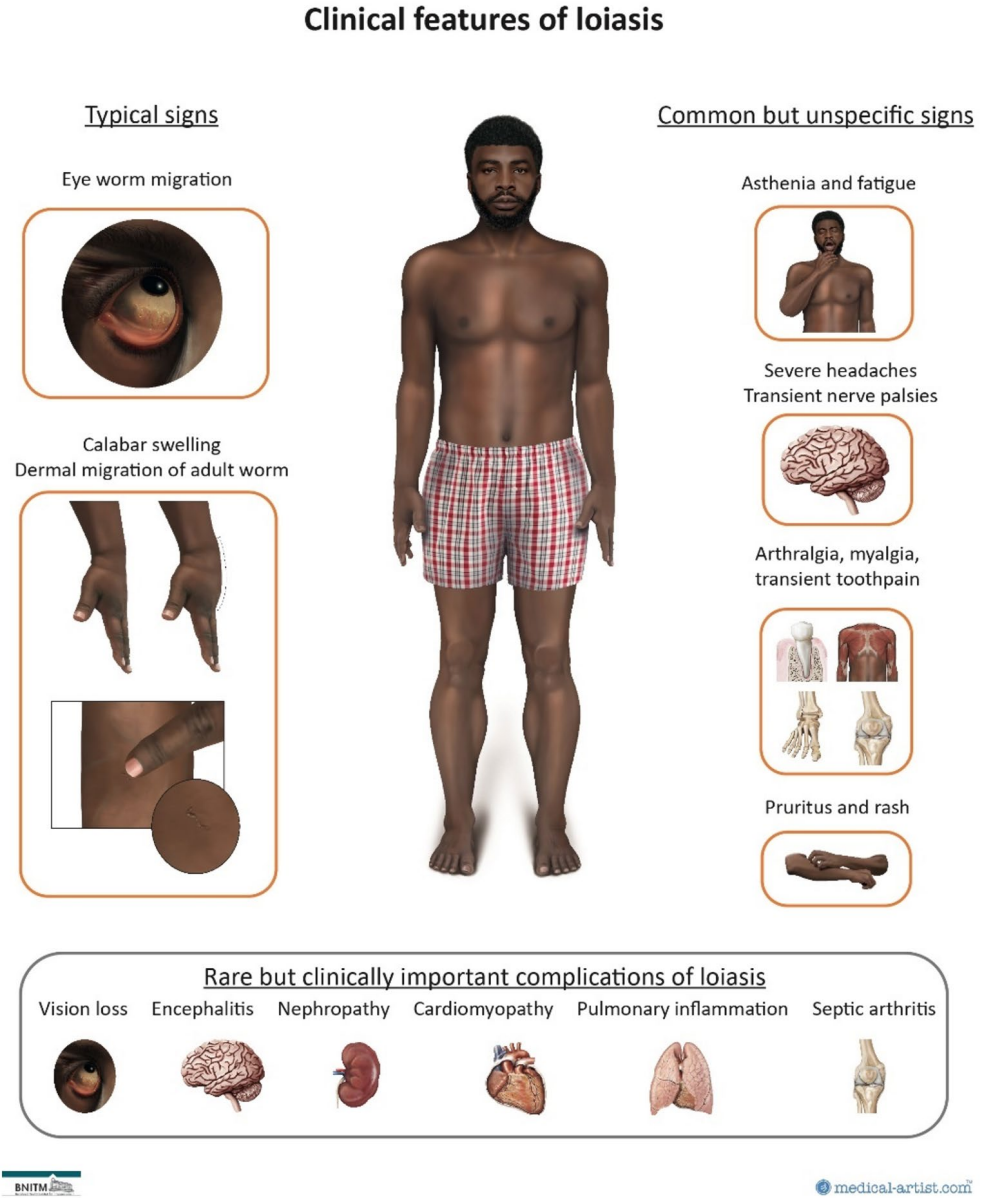
Clinical features of loiasis. Medical illustrations by Dr Joanna Butler 2024 (www.medical-artist.com). Rights to this figure are owned by medical-artist.com and the Bernhard Nocht Institute for Tropical Medicine. Permission was granted to use these graphics for this publication

Loiasis is characterized by varying clinical penetrance manifesting or not symptomatically. Besides an asymptomatic disease course, there are classical pathognomonic clinical symptoms, common but unspecific symptoms, and rare and occasionally life-threatening organ complications (Fig. 3) [2]. 

#### Typical clinical symptoms of loiasis

Typical clinical symptoms of migratory loiasis are to be considered pathognomonic when they appear in the context of a corresponding exposure in a known transmission region. The eponymous symptom of loiasis is the migration of the adult worm through the subconjunctival tissue of the sclera or eye lids (eye worm migration), which occurs at one stage in more than 80% of infected persons [2, 40]. While some patients report little or no symptoms of the eye worm migration, a substantial proportion of patients experiences pain, pruritus, or photophobia, making activity in daylight impossible [6]. The eye worm migration may therefore lead to a stabbing pain and a profound transient localized inflammatory reaction such as redness or swelling of the eye. The eye worm migration during which the adult worm becomes visible usually lasts between few hours and up to 7 days and most of the times fully resolves without any long-term sequelae. Worms may be removed surgically during the eye migration with a minor surgical procedure.

The second pathognomonic clinical feature of loiasis is localized, transient swelling mostly occurring on the extremities termed “Calabar swelling” [6]. Being the most frequent clinical manifestation, it may occur early in the disease course following infection. Calabar swelling is a transient, non-pitting, non-tender angioedema, caused by an allergic-immunological reaction of migrating adult worms. It occurs preferably adjacent to peripheral joints of the upper, more rarely, lower extremities, but can affect any region of the body including the face. It usually manifests for a period of a few hours to days and is most commonly highly pruritic and painful. Again, this classical symptom resolves spontaneously without permanent damage.

The superficial migration of the adult worm in the dermis may occasionally be observed presenting as a rapidly evolving serpentine efflorescence. Historically, administration of subtherapeutic doses of DEC has been associated with an increased frequency of dermal migration of *L. loa* due to its filarifugal properties [49]. In contrast to other helminth infections e.g. Larva migrans cutanea, the cutaneous migration of *L. loa* does not cause an inflammatory reaction, change of skin pigmentation or persistent serpiginous lesions.

#### Common but unspecific clinical symptoms of loiasis

Beside the typical symptoms of loiasis, patients commonly develop variable unspecific complaints. These include fatigue, myalgia, arthralgia, recurrent, severe headache (more than 50% of infected persons), temporary palsy of peripheral nerves (20%), paraesthesia, and transient tooth pain [6, 50]. Temporary disturbances or even loss of vision may be observed due to optic neuritis, as an exceptionally rare complication.

Dermatological symptoms including chronic pruritus, urticaria, rashes and temporary oedema are commonly reported. These unspecific symptoms often cause substantial chronic complaints representing an important part of the overall disease burden [6]. 

#### Rare, but clinically important complications of loiasis

While loiasis-associated organ damage is correlated with microfilarial load, clinically important complications of loiasis seem to occur only occasionally in a small subset of microfilaraemic patients. However, excess mortality is thought to be linked to these complications [4, 5]. Figure 3; Table 5 provide an overview of these organ complications.

One of the most important complications is loiasis-associated encephalopathy presenting with a broad range of clinical symptoms ranging from transient cognitive disorders to coma and death [51]. Encephalopathy occurs more frequently following administration of certain rapidly-acting microfilaricidal drugs such as diethylcarbamazine (DEC) and ivermectin. However, spontaneous episodes of loiasis-associated encephalopathy or encephalitis without prior treatment have been reported [52, 53]. Limited data from histopathological examination of fatal cases of encephalitis showed multiple microhaemorrhages, inflammation and cerebral oedema [54]. 

Renal changes such as haematuria and proteinuria have been described in patients with high microfilariae count and following therapy [55, 56]. Occasional reports of nephrotic syndrome or renal failure are indicative of their putative clinical importance. The pathophysiological mechanism of renal impairment appears to be linked to an immune complex deposition and membranoproliferative glomerulonephritis [56]. 

Endomyocardial fibrosis is among the most important cardiac complications linked to loiasis. While it is thought to present with an insidious onset, it may result in difficult-to-treat and ultimately life-threatening heart-failure. Pulmonary complications such as pleural effusions, ascites, calcified nodules of soft tissue, and other rare complications have been described in case reports [57]. 

The presence of microfilariae has been reported for almost all body fluids, including cerebrospinal fluid, ascites, pleural effusions, and synovial fluid. Their pathophysiological significance is still largely unknown [57]. Sonographic detection of hypoechogenic splenic lesions in patients with microfilaraemic loiasis are indicative for granulomatous reactions to filtered and dying microfilariae. Currently, these findings are thought to be of no clinical significance [58]. Similarly, microfilariae may be found in the placenta of pregnant women. So far, no link to inflammatory changes of the placenta or placental insufficiency has been reported for loiasis in pregnancy [59]. 

### Differential diagnosis of loiasis

Depending on the clinical or laboratory presentation of the patient several differential diagnoses have to be considered in case of unsuccessful parasitological confirmation of *L. loa* infection. The differential diagnoses of unspecific symptoms (urticaria, pruritus, joint swelling) are unsurprisingly broad. The evaluation of loiasis-associated laboratory disorders such as eosinophilia is similarly extensive. In general, it includes infections with helminths and specific other infectious pathogens, haematological and oncological diseases, autoimmune disorders, allergic and autoimmune diseases, and drug intolerance.

More specific symptoms such as Calabar swelling may be confounded with similar disease presentations caused by *Mansonella perstans* infection, or dermal reactions to larval migration of *Strongyloides stercoralis* or zoonotic hookworms. Moreover, several rheumatological, genetically predisposed and allergic disorders have to be included into the differential diagnoses.

The most prominent symptom of loiasis constitutes the ocular migration of the adult worm. Although eye worm migration in a person residing in a highly endemic region is considered pathognomonic, there are also descriptions of patients presenting with a migration of a worm through the eye in regions without known *L. loa* transmission. Considerations towards the differential diagnoses of the general observation of an eye worm migration are therefore of importance.

#### Differential diagnosis of eye worm migration

Several helminths endemic to different parts of the world, which may affect anatomical structures of the eye, may be considered as differential diagnoses of loiasis-associated eye worm migration [60]. The second most frequent helminth infection of the eye is dirofilariasis. *Dirofilaria repens* is the most important pathogen causing ocular manifestations, although other *Dirofilaria* species (*D. immitis*, *D. tenuis*) have occasionally been reported as causative pathogen. Similarly, several closely related zoonotic *Onchocerca* species have been described to cause ocular infection. Among other nematode species, *Thelazia spp.* have been repeatedly reported to affect the eye, while other species of the genera *Gnathostoma*,* Loaina*, *Acanthocheilonema* as well as *Toxocara*, *Baylisascaris*, *Angiostrongylus*,* Dracunculus* and *Trichinella* have so far been described only in single case reports. Trematodes are reported only rarely as pathogens affecting the eye including cases of infection with *Fasciola hepatica*,* Alaria* spp., *Philophthalmus lacrimosus.* Finally, cases of the cestodes *Spirometra* and *Spargana*,* Taenia*,* Coenurus* and *Echinococcus* spp. with ocular involvement have been reported occasionally in literature. Upon extraction, the morphological or molecular differentiation of *L. loa* from the other zoonotic helminth infections is usually straightforward.

## Diagnosis of *Loa loa* infection

Direct and indirect diagnostic methods are available for the diagnosis of *L. loa* infection [2]. While direct methods allow the proof of infection and the determination of the infecting pathogen at a species level, indirect methods may provide useful additional evidence supporting or refuting infection with *L. loa*. Since to date there are no sensitive methods for the direct detection of the adult worm, the diagnosis of occult loiasis is often challenging and must take not only the (indirect) biological findings but also the clinical signs and the epidemiological risk of infection into account to reliably assess for *L. loa* infection.

### Direct detection methods

#### Direct detection of microfilariae

In clinical practice, examination for microfilariae is usually performed by classical microscopy. Blood samples for loiasis diagnostics have to be taken around midday (10 am to 4 pm), as the microfilarial load is highest at this time of the day and by this means sensitivity of diagnostic assays is increased [61]. Capillary blood contains slightly higher microfilariae counts than venous blood, but this usually has no decisive therapeutic consequences in everyday clinical practice [62]. The blood can be examined directly and unstained for motile microfilariae. The snake-like movements can usually be detected quickly under low magnification [2]. 

The most important classical parasitological examination technique remains the preparation of a Giemsa stained thick blood smear from 50 µl of capillary blood but other rapid staining techniques have also been successfully employed [63]. Microscopic assessment of blood slides should be performed starting from low magnification (x10), the entire blood smear needs to be screened and higher magnification (x20, x40, x100 oil immersion) needs to be used in case of investigation of specific structures. The typical morphology (cell nuclei extending individually into the tail end, sheath present) and size (231–300 μm length, 6–8.5 μm thickness) of the microfilariae can thus be assessed visually. While the sheath is usually not well depicted in Giemsa stain, haematoxylin-based staining protocols provide a better visibility. In the absence of microfilariae detection in the thick blood smear, concentration methods such as Knott concentration technique or filtration of saponin-lysed blood are recommended to detect low levels of microfilaraemia [64].

Molecular detection techniques such as PCR and LAMP are established in some specialized laboratories leading to a higher sensitivity and specificity than classical microscopy. However, they have been developed for the detection of microfilariae in the blood and are not validated for the reliable diagnosis of the adult worm as in occult loiasis [65–67]. Other less common methods of direct microfilarial detection are microhaematocrit (e.g. QBC method) and buffy coat enrichment. Furthermore, a smartphone-based device (LoaScope or NTDScope, University of Berkley) has been developed to reliably detect hypermicrofilariaemic loiasis. It has been successfully tested in population-based control programs [68]. In general, however, the classical methods of microscopic species determination and quantification are until today best suited to guide clinical management.

In addition to the findings on the presence of microfilariae, their absolute number per mL of peripheral blood is important for the selection of appropriate treatment regimens (Table 4). To ensure reliable quantification, multiple (at least two) assessments of blood samples from different days should be obtained prior treatment initiation to avoid random fluctuations in the absolute microfilariae counts [61]. 

#### Direct detection of adult worms

The presence of adult worms cannot be directly assessed unless the worm is extracted from the body. Extraction is usually only successful during the short migration of the adult worm through the conjunctiva or superficial dermis. Extracted specimens may reliably be assessed for the species level by microscopical or molecular investigation. While some publications appear to show a high sensitivity for the detection of adult worms by molecular tools, these results have not yet been independently reproduced. Therefore, PCR is still not of clinical usefulness for the diagnosis of occult loiasis.

### Indirect detection methods

#### Serological procedures

Indirect evidence for *L. loa* infection may be obtained by detection of filarial-specific antibodies. For this purpose, antigen extracts of closely related filarial species *(*e.g. *L. loa*,* Dirofilaria spp.*,* or Acanthocheilonema viteae)* are usually employed and antibody titres (at times their IgG4 specific sub-fraction) are determined by ELISA. The relatively favorable sensitivity in patients from non-endemic regions contrasts to the low specificity towards other nematode infections. Serology is therefore often of only limited clinical usefulness in patients from endemic regions, as multiple life-time exposures to helminth antigens often result in cross-reactivity with helminth serology [69, 70]. In addition, active infections cannot be reliably distinguished by serology from previous exposures and infections which largely precludes its use in the follow-up of treated patients.

In addition to “in-house” serology, a commercially available rapid test detecting antibodies against Ll-SXP-1 has been evaluated [71]. While this test appears useful for epidemiological surveys in endemic areas, it is not suitable for use in individual patient management due to its limitations in sensitivity and specificity [69]. 

#### Indirect evidence from routine laboratory test results

Differential blood count and clinical chemistry parameters usually provide initial hints towards potential *L. loa* infection. In particular, the presence of eosinophilia with often extremely high absolute eosinophil cell counts is suggestive. Besides this, substantial elevation of IgE levels with at the same time unremarkable inflammatory markers (such as C-reactive protein) are common in loiasis before the start of treatment [33]. 

Routine haematology and biochemistry analysis including liver enzymes and creatinine is useful as baseline assessment before initiating therapy and to recognize potential contraindications of specific drugs. Similarly, the determination of proteinuria, blood eosinophilia, and IgE may become important for the further follow-up assessment of patients [72]. In the case of hypereosinophilia and hypermicrofilaraemia, further diagnostic work-up including echocardiography, and neurological examination should be considered depending on clinical presentation [2]. 

## Treatment

### General considerations about the treatment of loiasis

#### Indication for specific antifilarial treatment of loiasis

The indication for treating loiasis has not yet been sufficiently investigated based on objective evidence [2]. On one hand, a considerable somatic and psychological burden of disease is usually described in newly infected travellers, which may pathophysiologically be explained by a hyperresponsive immune response [37]. This, again, usually leads to a decision to treat. On the other hand, it is currently unclear whether and under which circumstances patients residing in highly endemic regions should receive antifilarial treatment. The decision to treat or not to treat a patients residing in an endemic region for loiasis should be based on the individual morbidity, the risk for serious complications, the risk of antifilarial treatment, and the probability of reinfection [2]. The availability of specific drugs, the at times long and complex treatment regimes, and locally available means to diagnose and manage treatment-associated complications are further factors that play an important role in the treatment decision [2]. 

Finally, specific contraindications of respective drugs must be considered. In addition to a high microfilarial load, the presence of onchocerciasis is a major contraindication for using DEC, as this may lead to Mazzotti reaction and irreversible vision loss [73, 74]. These reactions are caused by the rapid killing of *Onchocerca volvulus* microfilariae and consecutive hyper-inflammation. Prior to initiation of DEC therapy, onchocerciasis has therefore to be excluded in patients residing in regions where onchocerciasis is co-endemic or where its endemicity may be unclear. The use of albendazole is relatively contraindicated in the presence of active ocular or cerebral cysticercosis and should only be used with concomitant corticosteroid therapy to avoid complications of cysticercosis [75]. 

#### Considerations about treatment objectives and assessment of treatment outcome

The main goals of treatment for loiasis can be either individual cure of infection, resolution of clinical symptoms, or prevention of transmission, mostly in population-based control programs [2]. However, the assessment of treatment outcome may be challenging.

Clearance of peripheral microfilaraemia may be observed following treatment of microfilaraemic loiasis patients. However, the absence of microfilaraemia in itself does not confirm a cure of the infection as it does not prove the clearance of the adult worms. The complete clearance of adult worms may only be deduced from the evolution of indirect parameters, as the presence of adult *L. loa* worms cannot be assessed directly. Indirect parameters include the continued disappearance of peripheral microfilaraemia, resolution of clinical symptoms, normalisation of pre-existing eosinophilia and IgE levels, and at times a slow, often gradual reduction of filaria-specific antibodies over several years of follow-up. While these indirect markers are relatively specific in returning travellers, this is usually not the case in endemic regions due to multiple and repeated exposures to loiasis and other parasitic infections.

#### In-hospital versus outpatient treatment of loiasis

Due to the occurrence of serious adverse events in the treatment of loiasis, it is of high clinical importance to consider the setting of patient management. Hospitalisation of patients ensures rapid diagnosis and treatment of adverse drug reactions and allows early discontinuation or modification of treatment if necessary. While no general recommendation for or against in-patient or out-patient management can be provided, several considerations may be taken into account to decide on an appropriate setting and at times at least temporal hospitalisation at the initiation of therapy (Table 2).

**Table 2:** Considerations on inpatient versus outpatient management of loiasis

Microfilarial load	The risk of serious adverse events due to anthelmintic therapy is directly associated with microfilarial load.[76] Serious adverse events have been reported for microfilarial loads of 2,000 mf/mL blood and above. In general, the risk of adverse drug reactions is linked with pharmacodynamic characteristics of the respective drug against microfilariae. Serious adverse events have been reported repeatedly for DEC and ivermectin within the first 24 h after treatment initiation.[77] Hospitalisation of patients during treatment initiation with DEC or ivermectin is therefore to be considered. The overall duration of hospitalisation depends on the microfilarial load and the respective treatment regimen.
Hypermicrofilaraemia	Hypermicrofilaraemia represents an important risk factor for the occurrence of treatment associated serious adverse events.[76] Treatment initiation should therefore take place in an in-patient setting. Discharge depends on the tolerability of the treatment and the overall reduction of microfilarial load.
Occult loiasis	Treatment of occult loiasis is associated with significantly fewer risks than treatment of microfilaraemic loiasis. However, hospitalisation may be considered during treatment initiation with DEC.
DEC	DEC has the most rapid and powerful effect on microfilaraemia.[78, 79] Use of DEC may lead to anaphylactoid reactions and encephalopathy within a few hours. The use of sub-therapeutic doses of DEC is associated with filarifugal reactions, causing rapid and erratic migration of adult worms. Due to these features of DEC in the treatment of loiasis, hospitalisation of patients during treatment initiation should be considered.
Ivermectin	Ivermectin has a rapid effect on microfilariae and can therefore lead to serious adverse events which occur in most cases within the first 48 h. Hospitalisation should therefore be considered for the duration of treatment initiation with ivermectin.
Albendazole	Treatment of loiasis with albendazole is not associated with a rapid onset of adverse drug reactions, as albendazole has a delayed and only gradual effect on microfilariaemia while at the same time exerting some activity on adult worms.[80] Serious adverse events have been reported and occur usually after a delay of 14–28 days.

### Antifilarial drugs for the treatment of loiasis

In general, it must be noted that the antifilarial treatment of loiasis is always associated with a certain risk. Depending on the medication used, the microfilarial load, and the individual predisposition and comorbidities of the patient, several adverse events may occur which may range from acute anaphylactoid reactions, pruritus, allergic phenomena, to acute encephalitis, coma, and death. The indication for antifilarial therapy and the choice of treatment regimen must therefore be carefully reflected.

Historically, DEC is the only drug that has been developed specifically for the treatment of human filariasis [73]. It is the only drug with a proven macrofilaricidal effect therefore capable of inducing complete cure from infection with *L. loa*. Besides DEC, ivermectin and albendazole are commonly used antifilarial drugs in the treatment of loiasis (Tables 3 and 4).

**Table 3:** Activity of anthelmintic drugs against different life-cycle stages of *L. loa*

Drug	Activity on adult worms	Activity on microfilariae
DEC	++	+++
Ivermectin	?	+++
Albendazole	+	+
Doxycycline	-	-

#### DEC

DEC is an anthelmintic drug of the piperazine class. DEC has been the first-line treatment for loiasis for almost a century. Due to its rapid microfilaricidal effect, it may however lead to serious adverse events when treating loiasis. DEC is authorized in several countries but it is not marketed in Germany.

##### Indication

Treatment of occult and microfilaraemic loiasis. In the case of microfilaraemia > 2,000 mf/mL, DEC should not be used for treatment initiation. In this case the microfilarial load should first be reduced by other means (see below), to minimize the risk for the occurrence of serious to life-threatening complications.

##### Activity

Very rapid and potent activity against microfilariae. Substantial activity against adult worms. Cure rates when administered over a 4-week period are between 60 and 70%. Several cycles of DEC treatment may be necessary to achieve complete cure.

##### Side effects

Mazzotti reaction, which is particularly common in patients with onchocerciasis or disseminated *Strongyloides* infection, characterized by acute onset of itching, fever, headache, nausea, dizziness, fatigue, shortness of breath, cough, tachycardia, and proteinuria.

##### Contraindications

DEC may be associated with acute anaphylactic reactions in patients with high *L. loa* microfilariae load. Therefore, treatment with DEC requires close monitoring of patients. As the risk of allergic and encephalopathic side effects correlates in general with the microfilarial load, its use is relatively contraindicated in patients with microfilaraemia of more than 2,000 mf/ml. DEC is furthermore contraindicated in patients with active onchocerciasis. Furthermore, relative contraindications are the treatment of elderly patients, young children, patients with cardiac or renal disease, and chronic pathological, medicinal and dietary urine alkalisation. Caution should also be used when treating patients with a history of convulsions or epilepsy.

###### Pregnancy and lactation

relative contraindication. It is not known whether DEC passes into breast milk; breastfeeding is not recommended.

#### Ivermectin

Ivermectin is a macrolytic lactone that is used successfully and extensively as an antifilarial drug to control onchocerciasis and lymphatic filariasis. Due to its single-dose administration, it is used on a large scale in population-based control programs for onchocerciasis and lymphatic filariasis [68]. It interacts with glutamate-gated chloride channels of the parasite’s nerve and muscle cells. Due to its rapid and potent activity against *L. loa* microfilariae, ivermectin, similar to DEC, may lead to acute anaphylactic reactions and the occurrence of ivermectin-associated encephalitis.

##### Indication

Ivermectin is used for microfilaraemic loiasis with microfilaraemia between 2,000 mf/mL and 8,000 mf/mL. Risk for serious adverse events including encephalitis is strongly associated with microfilarial load. Therefore microfilaraemia > 8,000 mf/mL constitutes a relative contraindication. Treatment regimens combining ivermectin with albendazole (for microfilarial load < 8,000 mf/mL) may be considered.

##### Activity

Single dose ivermectin dramatically reduces the microfilarial load of *L. loa* by around 90%. So far, no convincing evidence of clinically relevant activity against adult worms has been demonstrated. Thus, the use of ivermectin is in itself not a curative regimen for loiasis. Ivermectin may be considered to reduce microfilaraemia from up to 8,000 mf/mL to enable subsequent curative therapy with DEC. Given the unclear benefit in alleviating clinical symptoms, which are mainly caused by the adult developmental stages of *L. loa*, the individual benefit of using ivermectin in treating loiasis is limited. A combination regimen of albendazole with ivermectin has shown promising efficacy at a single European centre [30, 81]. However, these findings were not confirmed in in a clinical trial in a highly endemic region [68]. Hence, to date the contribution of ivermectin to treatment success remains unclear.

##### Side effects

Risk of significant neurological complications in patients with high microfilarial load including potentially life-threatening encephalopathy that may initially present as altered mood, confusion, stupor, lethargy, headache, retinal and conjunctival haemorrhages, urinary incontinence or fever. Other side effects of ivermectin include pruritus, myalgia, urticarial exanthema, nausea, vomiting, diarrhoea, constipation, abdominal pain, dizziness, vertigo and rare cases of hepatitis.

##### Contraindications

Relative contraindication for use of ivermectin in patients with microfilarial load of more than 8,000 mf/mL due to the risk of treatment associated encephalitis. Ivermectin is not registered for use in children < 2 years of age or < 15 kg body weight.

##### Pregnancy and lactation

Use of ivermectin may only be considered after cautious risk-benefit analysis as animal experiments indicate reproductive toxicity. Breastfeeding should be suspended during the use of ivermectin, as high drug concentrations may be present in breast milk.

#### Albendazole

Albendazole is a broad-spectrum benzimidazole anthelmintic drug acting on parasite microtubules. Albendazole has a delayed, probably mainly indirect effect on microfilarial counts by reduction of fertility (reduction of microfilaraemia starts after about 2 weeks of treatment) and also appears to be effective against adult stages of *L. loa* in treatment regimens lasting several weeks [82]. To date, only a few hundred patients that underwent treatment for loiasis with prolonged albendazole regimens have been reported. Therefore no definitive conclusion can be drawn about the overall safety and efficacy of albendazole in treating loiasis [83]. 

##### Indication

Albendazole may be considered for use to reduce the microfilarial load due to its slow and gradual activity on microfilaraemia. However, single case reports of benzimidazole-associated encephalitis following loiasis therapy highlight the uncertainties of its safety in the treatment of loiasis. Albendazole shall be taken with food as fat uptake increases considerably its bioavailability.

##### Activity

Prolonged treatment courses with albendazole lead to a consistent, gradual reduction of the microfilarial load. Longer treatment regimens may lead to an adulticidal effect. The required duration of treatment is at least 3–4 weeks.

##### Side effects

Fever (1/10), leukopenia (1/100), anaemia (1/1000), pancytopenia (1/10,000), agranulocytosis, pruritus, urticaria, headache, dizziness (resulting in potentially impaired ability to drive), abdominal pain, diarrhoea, nausea, vomiting, increase in liver enzymes, reversible alopecia, Steven-Johnson syndrome (1/10,000). According to the approved summary of the product characteristics leaflet, liver function tests and blood counts should be performed prior to initiation and regularly during treatment.

##### Contraindications

known hypersensitivity to benzimidazole, children < 6 years of age, evidence or suspicion of active ocular or cerebral cysticercosis (history of seizures).

##### Pregnancy and lactation

Only when strongly indicated, animal model results indicate reproductive toxicity. Albendazole should not be used in breastfeeding women.

#### Doxycycline and other drugs without proven evidence in the treatment of loiasis

Doxycycline kills adult worms in the treatment of other human filariasis such as onchocerciasis. Its mechanism of action is based on the elimination of obligate *Wolbachia* spp. endosymbionts, resulting in slow sterilisation and subsequent death of the adult worms. As *L. loa* does not harbour *Wolbachia* endosymbionts, doxycycline does not affect *L. loa* infections and can therefore not be used to treat loiasis [84, 85]. 

Imatinib, a tyrosine kinase inhibitor developed for the treatment of chronic myeloid leukaemia, was found to be active against *L. loa*.[86] Although a reduction in microfilarial burden has been demonstrated in a clinical trial, its overall effect is insufficient from a clinical perspective. Imatinib therefore plays no role in the treatment of loiasis [87]. 

Moxidectin, a macrocyclic lactone closely related to ivermectin, is currently undergoing phase II clinical trials to evaluate its safety and efficacy in the treatment of loiasis [88]. However, to date efficacy and safety are not yet sufficiently established for the treatment of loiasis.

### Specific patient populations

#### Children

From an epidemiological perspective, children are usually less affected by *L. loa* infections than adults, both in endemic regions and among travellers [6, 36]. To date there are no reports on specific clinical or therapeutic characteristics of paediatric loiasis. Treatment, therefore, follows the same principles as for adults. Age- and weight-related contraindications for respective drugs must be considered.

#### Pregnancy and lactation

Loiasis commonly affects pregnant women as the infection lasts for many years, often lifelong in high-transmission regions. Microfilariae may be detectable in the placenta or the umbilical cord’s blood but do not appear to cause inflammation, placental insufficiency or other clinically important pathologies [59]. To date there is no clear evidence for adverse birth outcomes due to loiasis. Vertical or peri-partal transmission of *L. loa* from the mother to the child has not been reported. Given the often-uncertain risks of anthelmintic therapy during pregnancy, treatment should in general be postponed until after delivery and possibly after breastfeeding. In case of substantial clinical or psychological burden due to loiasis in an individual pregnant patient, benefits and risks of treatment have to be discussed with the patient in light of the lack of systematic evidence.

#### Elderly patients and patients with co-morbidities

The risk of clinically important adverse drug reactions of DEC may be increased in patients with pre-existing cardiac and renal morbidity. Prolongation of DEC’s half-life has been reported in patients with renal insufficiency or in conditions with chronic urine alkalisation. A dose reduction is therefore recommended in patients with renal insufficiency. Caution is also advised in patients with a history of seizures, as seizure threshold may be lowered.

Albendazole should be used with caution and monitored closely in patients with pre-existing hepatic impairment and in elderly patients.

### Non-medical treatment options for loiasis

#### Surgical removal of adult worms

Surgical removal can be performed during the migration of the adult worm through the eye or the superficial cutis [89]. These migrating worms can be removed by incision and extraction under sterile conditions to avoid secondary bacterial infection. Extraction has to be performed within a short period of time to avoid the disappearance of the worm in the adjacent soft tissue. Extraction may at times be curative in travellers with single worm infections. However, patients in high transmission regions are usually infected with multiple adult worms, so that extraction of a single adult worm is not curative and therefore constitutes not a therapeutic priority.

#### Apheresis

For several decades apheresis has been described as a safe method to gradually and safely reduce high levels of circulating microfilariae from peripheral blood [90]. Mechanical reduction of the microfilarial load, which usually requires several cycles, is not curative as it does not clear the adult worms. However, it may constitute a safe option for substantial reduction of microfilarial load prior to initiation of a drug-based curative treatment approach.

### Drug regimens to treat loiasis

The appropriate choice of a specific treatment regimen depends on several factors and should be made by a specialist experienced in tropical medicine. In addition to the drugs’ local availability and the patient’s specific contraindications, the microfilarial load is of decisive importance when choosing a treatment regimen. It needs to be emphasised here again that the microfilarial load should be measured on several days during the midday hours (10 a.m. − 4 p.m.) to obtain reliable microfilaraemia estimates, as there is a strong periodicity of microfilaraemia over the 24 h of the day (see Table 4).

**Table 4:** Antifilarial treatment of loiasis

Occult loiasis	DEC (first-line treatment)	9 mg/kg in three divided doses daily for 21 days; more than one treatment regimen of 21 days may be necessary as cure rate is between 60–70% per cycle
	Albendazole (alternative treatment option)	400 mg twice daily for 4 weeks; cure rate is unclear; Follow-up of transaminase and haematology during treatment are recommended
	Albendazole-ivermectin (alternative treatment option)	400 mg albendazole twice daily for 4 weeks followed by a single dose of 150–200 µg/kg ivermectin; Contradictory reports about cure rates; Follow-up of transaminases and haematology during treatment are recommended
**1–2**,**000 mf/mL**	DEC (first-line treatment)	Slow titration: 50 mg DEC once on Day 1 of treatment, 50 mg DEC three times on Day 2 of treatment, 100 mg DEC three times on Day 3 of treatment, 9 mg/kg DEC per day, divided in three doses, up to Day 21
	Albendazole (alternative treatment option)	400 mg albendazole twice daily for 4 weeks followed by a single dose of 150–200 µg/kg ivermectin; Contradictory reports about cure rates; Follow-up of transaminases and haematology during treatment are recommended
	Albendazole-ivermectin (alternative treatment option)	400 mg albendazole twice daily for 4 weeks followed by a single dose of 150–200 µg/kg ivermectin; Contradictory reports about cure rates; Follow-up of transaminases and haematology during treatment are recommended
**2**,**000–8**,**000 mf/mL**	Albendazole (first-line treatment)	400 mg albendazole twice daily for 4 weeks; this regimen is used either as stand-alone regimen or it is administered to reduce microfilarial load below 2,000 mf/mL before administration of DEC (see above); Due to the limited number of clinical trials evaluating this regimen, the overall safety of this treatment regimen remains unclear; Follow-up of transaminases and haematology during treatment are recommended
	Ivermectin (first-line treatment	150–200 µg/kg single dose; this regimen is administered to reduce the microfilarial load below 2,000 mf/mL before administrating DEC (see above);
	Albendazole-Ivermectin (alternative treatment option)	400 mg albendazole twice daily for 4 weeks followed by a single dose of 150–200 µg/kg ivermectin; This regimen is administered either as a stand-alone regimen or it is administered to reduce microfilarial load. Contradictory reports about cure rates; Due to the limited number of clinical trials evaluating this regimen, the overall safety of this treatment regimen remains unclear. Follow-up of transaminases and haematology during treatment are recommended.
**8**,**000–30**,**000 mf/mL**	Apheresis (potential treatment option)	Mechanic reduction of the microfilarial load in several cycles of apheresis to less than 8,000 mf/mL prior to administration of ivermectin or albendazole (see above). Alternatively, reduction of microfilarial load below 2,000 mf/mL prior to the administration of DEC (see above);
	Albendazole (potential treatment option)	400 mg albendazole once or twice daily for 4 weeks; this regimen is administered to reduce the microfilarial load below 8,000 mf/mL prior to the administration of ivermectin, or to reduce the microfilarial load below 2,000 mf/mL prior to the administration of DEC (see above); Due to limited number of clinical trials evaluating this regimen, the overall safety of this treatment regimen (including benzimidazole induced encephalitis) remains unclear; Follow-up of transaminases and haematology during treatment are recommended.
**> 30**,**000 mf/mL**	Apheresis (first-line treatment)	Mechanic reduction of the microfilarial load in several cycles of apheresis to less than 8,000 mf/mL prior to the administration of ivermectin or albendazole (see above). Alternatively, reduction of the microfilarial load below 2,000 mf/mL prior to the administration of DEC (see above);
	Albendazole (alternative treatment option)	Due to the limited number of clinical trials evaluating this regimen, the overall safety of this treatment regimen (including benzimidazole induced encephalitis) remains unclear; Follow-up of transaminases and haematology during treatment are recommended; albendazole should only be administered to hypermicrofilaraemic individuals based on a cautious risk and benefit analysis.

DEC: Contraindication in patients who are co-infected with onchocerciasis

Albendazole: Relative contraindication when suffering from ocular or cerebral cysticercosis

The following treatment regimens are recommended, depending on the microfilarial load and taking into account individual contraindications:

### Adjunct treatments for loiasis

Antihistamines and corticosteroids may be used during treatment initiation to reduce the severity of adverse events. However, these adjunctive treatments are considered not to alter the overall risk or seriousness of potentially life-threatening serious adverse events, including encephalopathy [10]. 

The monoclonal anti-IL-5 antibody reslizumab was evaluated in a clinical trial for its potential to reduce eosinophilia [91]. While a significant reduction in absolute eosinophil count without a delay of reduction of microfilarial load was observed, a clinically relevant benefit from this treatment has not been concluded. Thus, reslizumab therapy is not currently recommended for the treatment of loiasis.

### Treatment of clinical signs, symptoms, and complications of loiasis

The clinical manifestations of loiasis cause a significant subjective disease burden for affected patients [6]. Symptoms often persist for decades or even life-long in patients residing in high transmission regions due to the longevity of the adult worm and frequent re-infections. Clinical symptoms are often even more pronounced in returning travellers [37]. The only causal treatment of these symptoms constitutes antifilarial treatment to kill the adult worm. To date, there are no evidence-based treatment recommendations for many of the clinical symptoms of loiasis. However, considerations on the appropriate reduction or relief of clinical symptoms and the management of potentially life-threatening complications are of medical importance and shall be addressed here (see Table 5).

**Table 5:** Treatment of clinical signs, symptoms, and complications of loiasis

Typical clinical signs of loiasis	
Eye worm migration	During the eye worm migration, the adult worm is usually present in the conjunctiva of the eyelid or the sclera. Surgical removal of the worm is straightforward but must be performed without delay before the worm continues its migration, disappearing in the adjacent soft tissue. Removal is curative only in exceptional cases (travellers from non-endemic areas).
Calabar swelling	Transient swellings are caused by angioedema and are often pruritic and painful. Symptomatic treatment with non-steroidal anti-inflammatory drugs and antihistamines may be considered. The only causal treatment constitutes curative treatment of the adult worm.
Dermal migration of the adult worms	The occasional migration of the adult worm through the superficial dermis does usually not lead to substantial clinical symptoms. However, the adult worm may be removed from this location easily. Due to the presence of multiple adult worms, this removal is often not curative.
Cephalea, asthenia, myalgia, arthralgia	These unspecific symptoms often cause significant disease burden. Symptomatic treatment with antihistamines and antiphlogistic drugs may be tried for some time. The only causal treatment constitutes curative treatment of the adult worm.
Pruritus, urticaria, and other dermal efflorescences	Dermatological symptoms occur frequently and often over a long period of time. Symptomatic treatment with antihistamines may be tried. The only causal treatment constitutes curative treatment of the adult worm.
Encephalopathy and encephalitis	The onset of encephalopathy is often characterised by initial, unspecific signs, including behavioural change, agitation and changes in mood.[51] Signs of encephalitis include the presence of high fever, markedly elevated inflammatory parameters, and altered consciousness, which can progress to deep coma and death. There is currently no evidence-based treatment for loiasis-associated-encephalitis. Antifilarial treatment should be stopped immediately if symptoms occur. The use of high-dose corticosteroids is controversial: While the use of corticosteroids has been discouraged for sub-Saharan African settings due to safety concerns about secondary infections (e.g. strongyloidiasis), their use in a high resource setting may be considered based on the presumed pathophysiology of acute cerebral inflammation. In addition to supportive intensive care, important differential diagnoses such as bacterial meningitis, other forms of sepsis or hyperinflammation must be evaluated.
Transient paresis	The pathophysiology of transient paresis is thought to be mediated by an inflammatory, transient neuronal dysfunction, which usually resolves completely over time.[57] Therefore, no specific therapeutic measures are required.
Cardiac complications	Endomyocardial fibrosis is a chronic restrictive heart disease that leads to progressive chronic heart failure.[92, 93] Endomyocardial fibrosis is characterised by fibrotic thickening of the endocardium and myocardium of one or both ventricles, resulting in restrictive ventricular filling. Epidemiologically, there is a strong association with hypereosinophilia, which is often caused by long-lasting infections with helminths including *L. loa*. Genetic predisposition of patients also seems to play an important role in the pathophysiology of endomyocardial fibrosis. Therapeutically, the treatment of the cause of hypereosinophilia is of crucial importance. Cardiac surgery may be discussed in advanced endomyocardial fibrosis depending on its individual morphology.
Arthritis	Septic arthritis is a rare presentation of loiasis. It may be diagnosed by the detection of microfilariae in the synovial fluid. Systemic antiphlogistic drugs in combination with curative treatment of loiasis are recommended.
Ocular complications	The rare migration of *L. loa* into anatomical structures of the bulbus oculi constitutes an ophthalmological emergency. Immediate ophthalmic surgery is usually required to prevent permanent damage to vision. Transient visual impairment may be caused by inflammatory involvement of the optic nerve and often resolves spontaneously.
Nephropathy	Up to one third of patients have relevant haematuria and proteinuria, which are probably mediated by immune complex deposition.[55] Clinically relevant renal insufficiency is however rare and has been reported only in single case reports.
Splenic lesions	Hypodense splenic lesions are described in a small proportion of patients at ultrasound examination. This finding seems to be related to microfilaraemia and may constitute the granulomatous inflammatory reaction of dying microfilariae.[58] The lesions seem to be of no specific clinical importance. However, it is important to recognise this sign of loiasis to avoid unnecessary surgery (splenectomy) or invasive investigations.

### Follow-up of loiasis

Since loiasis is a chronic infection, prolonged follow-up of patients is necessary to assess potential complications and to monitor treatment outcomes after curative therapy.

#### Treatment evaluation

The overall reduction in microfilarial load is a simple parameter for assessing the microfilaricidal effect of anthelmintic treatment regimens. Depending on the respective drug, the maximum effect on microfilarial load may be expected within the first 4 weeks. If microfilaraemia persists over several months after treatment, it is therefore likely that the treatment has failed to clear active infection.

It is not possible to ascertain treatment success against adult worms in human infections, as there is currently no means to detect the adult stages of *L. loa* directly. The death of adult worms is likely to take several days to weeks after treatment initiation. Similarly, it may take several months until the dead adult worm has been cleared from the tissue. Successful treatment is usually accompanied by a transient increase in eosinophilia and IgE levels over the first few weeks, followed by subsequent normalisation of these two laboratory parameters. Without further clinical symptoms and normalisation of eosinophil count and IgE levels, complete cure from active *L. loa* infection can be assumed. Follow-up examinations at intervals of several months up to at least one year after the start of therapy are recommended to reliably evaluate the therapeutic success over time [30, 33, 83]. 

Specific filarial serology, which is of diagnostic importance in the initial diagnosis of travellers, plays only a minor role in follow-up. After successful treatment, filaria-specific antibody titres usually reduce only gradually over long periods of time, often over several years. At the same time, anti-filarial titres may persist without suggesting treatment failure. Therefore, the follow-up of filarial serology is not routinely recommended and, if at all, may only provide indirect evidence for cure or treatment failure.

It is not uncommon that the first treatment cycle may not lead to complete cure [79, 80]. It is estimated, that cure rates of the standard 3-week DEC regimen ranges between 60 and 70%. Repetitive treatment cycles of DEC are at times necessary to fully cure patients of *L. loa* infection [79]. Even in the absence of specific drug resistance of *L. loa* to DEC, an alternative regimen (e.g. prolonged albendazole or albendazole-ivermectin combination therapy; see Table 4) may be considered in case of persistence of infection several months after treatment [80]. Patients should be informed of the not uncommon necessity for multiple cycles prior to treatment initiation. In case of presence of complications of loiasis, further follow-up is required even after successful curative treatment.

#### Follow-up of patients without curative treatment for loiasis

In case that loiasis is not treated curatively (either on purpose withholding therapy or in case of unsuccessful therapy), particular attention should be paid to the possible long-term consequences of hypereosinophilia. 6-12-monthly follow-up examinations including echocardiography and, if necessary, other imaging techniques are indicated to appropriately detect and treat chronic long-term consequences of hypereosinophilia at an early stage.

### Prevention and prophylaxis of loiasis

*L. loa* is an only occasionally imported travel-related infectious disease. This fact is due to multiple reasons: The rural transmission cycle of loiasis does not coincide with typical tourist destinations; transmission occurs only during the day in forest and savannah regions of endemic countries and transmission does not appear to be very efficient, so that in most cases only prolonged exposure is associated with infection. At the same time, patients who have already had a *L. loa* infection are often at a disproportionate risk of acquiring another new infection, as these individuals frequently travel to the high transmission regions due to family (visiting friends and relatives) or professional reasons (missionaries, researchers, forest workers, military, etc.) [30]. Repeated and/or prolonged exposure in transmission regions therefore poses a relevant risk of re-infection with *L. loa*.

Several preventive tools are available to protect oneself against infection. Firstly, bites by the diurnal deerflies of the genus *Chrysops* should be avoided. This may be achieved by avoiding exposure in the relevant regions, by wearing long and light-coloured clothing and by applying repellents to the skin and cloths. Although no systematic studies on the effectiveness of repellents against *Chrysops silacea* and *Chrysops dimidiata* are available to date, a certain degree of protection may be assumed by standard repellents. Other protective measures against *Chrysops* bites, such as mosquito-net hats, caps or helmets, as well as protection by bednets when outdoors and screening of houses and apartments with nets on windows and doors, should be considered to prevent bites by deerflies.

In case of particularly high risk, such as multiple new infections after successful treatment due to repeated high-risk exposure in endemic areas, chemoprophylaxis for *L. loa* may be considered for individual cases. The effectiveness of DEC for the prophylaxis of loiasis was first described in animal models [94]. In a later randomized, controlled clinical trial, weekly oral administration of 300 mg DEC showed high protective efficacy (100% protection against clinical disease) in US adults traveling to high-transmission regions for long-term stays [95]. This chemoprophylaxis regimen, which was taken over a period of 2 years in the clinical trial, may therefore be considered for individual cases. However, detailed information on the off-label use of this drug, which is not approved for this indication in Germany, has to been provided to the traveller.

Regular laboratory follow-up appears to be useful in the long-term use of DEC to ensure tolerability and safety of the chemoprophylaxis regimen. Compared to placebo, nausea was described more frequently in the DEC group in this study. Relevant contraindications of DEC (onchocerciasis, active *L. loa* infection with microfilariae load > 2,000/mL, caution of accumulation in case of prolonged alkalinised urine) must be taken into account, particularly when considering chemoprophylaxis in individuals from endemic regions.

### Loiasis and blood transfusions

Blood transfusions are in general not necessary to treat *L. loa* infection as loiasis does not lead in itself to clinically important anaemia. However, in endemic regions with high prevalence of infection, the decision whether microfilaraemic individuals may serve safely as blood donors is commonly encountered as medical conundrum.

In the case of *L. loa* microfilaraemic blood donors, a risk-benefit analysis needs to be considered [96]. On the one hand, transfusion of microfilariae may cause secondary activation of the immune system of the blood transfusion recipient, leading to eosinophilia, increase of IgE and potential transient clinical symptoms including pruritus and urticaria. On the other hand, no patent infection is caused by administration of microfilaraemic blood, as microfilariae cannot multiply themselves within the human host. Consecutively microfilaraemia clears within several days or weeks without further medical intervention. Importantly, blood transfusions cannot transmit adult worms of *L. loa* due to their extravascular location in the soft tissues of the human host, thus precluding the possibility of blood-borne transmission of infection [2]. 

In summary, it may therefore be concluded that blood transfusions from blood donors without *L. loa* microfilaraemia are preferred. The presence of microfilariae in donor blood may, however, be considered of only minor medical risk for the recipient and must at all means not preclude the administration of a lifesaving blood transfusion.

## Data Availability

No datasets were generated or analysed during the current study.

## References

[1] Grove DI. A history of human helminthology. CABI Publishing; 1990.

[2] Ramharter M, Butler J, Mombo-Ngoma G, Nordmann T, Davi SD, Zoleko Manego R. The African eye worm: current understanding of the epidemiology, clinical disease, and treatment of loiasis. Lancet Infect Dis. 2024. 10.1016/s1473-3099(23)00438-3. (Epub 20231016. PubMed PMID: 37858326). 37858326 10.1016/S1473-3099(23)00438-3

[3] Metzger WG, Mordmuller B. Loa loa-does it deserve to be neglected? Lancet Infect Dis. 2014;14(4):353–7. 10.1016/S1473-3099(13)70263-9. (Epub 20131212. PubMed PMID: 24332895). 24332895 10.1016/S1473-3099(13)70263-9

[4] Hemilembolo MC, Niama AC, Campillo JT, Pion SD, Missamou F, Whittaker C, et al. Excess mortality Associated with Loiasis: confirmation by a New Retrospective Cohort Study conducted in the Republic of Congo. Open Forum Infect Dis. 2023;10(3):ofad103. 10.1093/ofid/ofad103. (Epub 20131212. PubMed PMID: 36968967; PubMed Central PMCID: PMC10034755). 36968967 10.1093/ofid/ofad103 PMC10034755

[5] Chesnais CB, Takougang I, Paguele M, Pion SD, Boussinesq M. Excess mortality associated with loiasis: a retrospective population-based cohort study. Lancet Infect Dis. 2017;17(1):108–16. 10.1016/s1473-3099(16)30405-4. (Epub 20161021. PubMed PMID: 27777031). 27777031 10.1016/S1473-3099(16)30405-4

[6] Epub 20200622. PubMed PMID: 32585133).

[7] Fauci AS, Braunwald E, Isselbacher KJ, Wilson JD, Martin JB, Kasper DL et al. Harrison’s Principles of Internal Medicine. 14th edition. McGrawHill. 1997.

[8] Neumayr A. Antiparasitic Treatment Recommendations: a practical guide to clinical parasitology. tredition; 2018.

[9] Farrar J, Hotez PJ, Junghanss T, Kang G, Lalloo DG, White NJ et al. Manson’s Trop Dis. 2023:758–61.

[10] Boussinesq M. Loiasis: new epidemiologic insights and proposed treatment strategy. J Travel Med. 2012;19(3):140–3. 10.1111/j.1708-8305.2012.00605.x. 22530819 10.1111/j.1708-8305.2012.00605.x

[11] Ramharter M, Schlabe S, Huebner MP, Michelitsch P, Kurth F, Bélard S et al. S1-Handlungsempfehlung: Diagnostik und Therapie der Loiasis (Afrikanischer Augenwurm). 2024. chrome-extension://efaidnbmnnnibpcajpcglclefindmkajhttps://register.awmf.org/assets/guidelines/042-011l_S1_Diagnostik-Therapie-Loiasis-Afrikanischer-Augenwurm__2024-06.pdf. Accessed 26 Jun 2024.

[12] Ramharter M, Schlabe S, Huebner MP, Michelitsch P, Kurth F, Bélard S et al. Tabelle zur Erklärung von Interessen und Umgang mit Interessenkonflikten. 2024. https://register.awmf.org/assets/guidelines/042_D_Ges_fuer_Tropenmedizin_und_Int_Gesundheit/042-011i_S1_Diagnostik-Therapie-Loiasis-Afrikanischer-Augenwurm__2024-06.pdf. Accessed 26 Jun 2024.

[13] e.V. AdWMF. Stufenklassifikation nach Systematik. https://www.awmf.org/regelwerk/stufenklassifikation-nach-systematik. Accessed 23 Sep 2024.

[14] Oldroyd H. The horse-flies (Diptera: Tabanidae) of the Ethiopian Region. British Museum (NH); 1952.

[15] Kilarski WW, Martin C, Pisano M, Bain O, Babayan SA, Swartz MA. Inherent biomechanical traits enable infective filariae to disseminate through collecting lymphatic vessels. Nat Commun. 2019;10(1):2895. 10.1038/s41467-019-10675-2. (PubMed PMID: 31263185; PubMed Central PMCID: PMC6603047). 31263185 10.1038/s41467-019-10675-2 PMC6603047

[16] Eberhard ML, Orihel TC. Development and larval morphology of Loa loa in experimental primate hosts. J Parasitol. 1981;67(4):556–64. (PubMed PMID: 7196445). 7196445

[17] Niamsi-Emalio Y, Nana-Djeunga HC, Chesnais CB, Pion SDS, Tchatchueng-Mbougua JB, Boussinesq M, et al. Unusual localization of blood-borne Loa loa Microfilariae in the skin depends on Microfilarial Density in the blood: implications for Onchocerciasis diagnosis in coendemic areas. Clin Infect Dis. 2021;72(Suppl 3):S158–64. 10.1093/cid/ciab255. (PubMed PMID: 33909066; PubMed Central PMCID: PMC8201578). 33909066 10.1093/cid/ciab255 PMC8201578

[18] Cox FE. History of human parasitology. Clin Microbiol Rev. 2002;15(4):595–612. 10.1128/CMR.15.4.595-612.2002. Erratum in: Clin Microbiol Rev. 2003. (PubMed PMID: 12364371; PubMed Central PMCID: PMC126866). 10.1128/CMR.15.4.595-612.2002 PMC126866 12364371

[19] Zoure HG, Wanji S, Noma M, Amazigo UV, Diggle PJ, Tekle AH, et al. The geographic distribution of Loa loa in Africa: results of large-scale implementation of the Rapid Assessment Procedure for Loiasis (RAPLOA). PLoS Negl Trop Dis. 2011;5(6):e1210. 10.1371/journal.pntd.0001210. (Epub 20110628. PMID: 21738809; PMCID: PMC3125145). 21738809 10.1371/journal.pntd.0001210 PMC3125145

[20] Duke BO. Studies on the biting habits of Chrysops. IV. The dispersal of Chrysops silacea over cleared areas from the rain-forest at Kumba, British Cameroons. Ann Trop Med Parasitol. 1955;49(4):368–75. (PubMed PMID: 13283506). 13283506

[21] Duke BO. The transmission of loiasis in the forest-fringe area of the British Cameroons. Ann Trop Med Parasitol. 1954;48(4):349–. 10.1080/00034983.1954.11685634. (PubMed PMID: 13229269). 55. 13229269 10.1080/00034983.1954.11685634

[22] Wanji S, Akotshi DO, Mutro MN, Tepage F, Ukety TO, Diggle PJ, et al. Validation of the rapid assessment procedure for loiasis (RAPLOA) in the Democratic Republic of Congo. Parasit Vectors. 2012;5:25. 10.1186/1756-3305-5-25. (PubMed PMID: 22300872; PubMed Central PMCID: PMC3292485). 22300872 10.1186/1756-3305-5-25 PMC3292485

[23] Wanji S, Tendongfor N, Esum M, Yundze SS, Taylor MJ, Enyong P. Combined utilisation of Rapid Assessment procedures for Loiasis (RAPLOA) and onchocerciasis (REA) in rain forest villages of Cameroon. Filaria J. 2005;4(1):2. 10.1186/1475-2883-4-2. (PubMed PMID: 15817124; PubMed Central PMCID: PMC1090603). 10.1186/1475-2883-4-2 PMC1090603 15817124

[24] Orihel TC, Lowrie RC Jr. Loa loa: development to the infective stage in an American deerfly, Chrysops atlanticus. Am J Trop Med Hyg. 1975;24(4):610–5. 10.4269/ajtmh.1975.24.610. (PubMed PMID: 1057379). 1057379 10.4269/ajtmh.1975.24.610

[25] Whittaker C, Walker M, Pion SDS, Chesnais CB, Boussinesq M, Basanez MG. The Population Biology and Transmission dynamics of Loa loa. Trends Parasitol. 2018. 10.1016/j.pt.2017.12.003. (Epub 20180110. PubMed PMID: 29331268). 10.1016/j.pt.2017.12.003 29331268

[26] Garcia A, Abel L, Cot M, Ranque S, Richard P, Boussinesq M, et al. Longitudinal survey of Loa loa filariasis in southern Cameroon: long-term stability and factors influencing individual microfilarial status. Am J Trop Med Hyg. 1995;52(4):370–5. 10.4269/ajtmh.1995.52.370. (PubMed PMID: 7741181). 7741181 10.4269/ajtmh.1995.52.370

[27] Moutongo Mouandza R, Mourou JR, Moutombi Ditombi B, Roger Sibi Matotou H, Ekomi B, Bouyou-Akotet MK, et al. Sociodemographics, clinical factors, and Biological Factors Associated with Loiasis in endemic onchocerciasis areas in Southern Gabon. Am J Trop Med Hyg. 2023. 10.4269/ajtmh.22-0558. (PubMed PMID: 37339766; PubMed Central PMCID: PMC10551092). 37339766 10.4269/ajtmh.22-0558 PMC10551092

[28] Pongui Ngondza B, Koumba Lengongo JV, Mickala P, M’Bondoukwé NP, Ndong Ngomo JM, Moutombi Ditombi BC, et al. Prevalence and risk factors for blood filariasis among HIV-infected adults in Gabon, Central Africa: a pilot study. Trans R Soc Trop Med Hyg. 2022;116(11):1015–21. 10.1093/trstmh/trac034. (PubMed PMID: 35474144). 35474144 10.1093/trstmh/trac034

[29] Antinori S. Imported Loa loa filariasis: three cases and a review of cases reported in non-endemic countries in the past 25 years. Int J Infect Dis. 2012;16(9):e649–62. 10.1016/j.ijid.2012.05.1023. (Epub 20120710. PubMed PMID: 22784545). 22784545 10.1016/j.ijid.2012.05.1023

[30] Nordmann T, Ruge J, Tappe D, Ramharter M. Imported loiasis at a clinical reference center in Germany: a retrospective case series. Am J Trop Med Hyg. 2024;in press:521–5. 10.4269/ajtmh.24-0022. (PubMed PMID: 38981492; PubMed Central PMCID: PMC11376174). 10.4269/ajtmh.24-0022 PMC11376174 38981492

[31] Bouchaud O, Matheron S, Loarec A, Dupouy Camet J, Bouree P, Godineau N, et al. Imported loiasis in France: a retrospective analysis of 167 cases with comparison between sub-saharan and non sub-saharan African patients. BMC Infect Dis. 2020;20(1):63. 10.1186/s12879-019-4740-6. (PubMed PMID: 31959110; PubMed Central PMCID: PMC6971866). 31959110 10.1186/s12879-019-4740-6 PMC6971866

[32] Gantois N, Rapp C, Gautret P, Ficko C, Savini H, Larreché S, et al. Imported loiasis in France: a retrospective analysis of 47 cases. Travel Med Infect Dis. 2013;11(6):366–. 10.1016/j.tmaid.2013.08.005. (Epub 20130829. PubMed PMID: 24035648). 73. 24035648 10.1016/j.tmaid.2013.08.005

[33] Gobbi F, Postiglione C, Angheben A, Marocco S, Monteiro G, Buonfrate D, et al. Imported loiasis in Italy: an analysis of 100 cases. Travel Med Infect Dis. 2014;12. 10.1016/j.tmaid.2014.07.004. (Epub 2014081. PubMed PMID: 25131142). 6 Pt B):713-7. 10.1016/j.tmaid.2014.07.004 25131142

[34] Bottieau E, Huits R, Van Den Broucke S, Maniewski U, Declercq S, Brosius I, et al. Human filariasis in Travelers and migrants: a retrospective 25-year analysis at the Institute of Tropical Medicine, Antwerp, Belgium. Clin Infect Dis. 2022;74(11):1972–8. 10.1093/cid/ciab751. (PubMed PMID: 34463732). 34463732 10.1093/cid/ciab751

[35] Saito M, Armstrong M, Boadi S, Lowe P, Chiodini PL, Doherty T. Clinical features of Imported Loiasis: a Case Series from the hospital for Tropical diseases, London. Am J Trop Med Hyg. 2015;93(3):607–. 10.4269/ajtmh.15-0214. (Epub 20150622. PubMed PMID: 26101271; PubMed Central PMCID: PMC4559705). 11. 26101271 10.4269/ajtmh.15-0214 PMC4559705

[36] Lipner EM, Law MA, Barnett E, Keystone JS, von Sonnenburg F, Loutan L, et al. Filariasis in travelers presenting to the GeoSentinel Surveillance Network. PLoS Negl Trop Dis. 2007;1(3):e88. 10.1371/journal.pntd.0000088. (PubMed PMID: 18160987; PubMed Central PMCID: PMC2154385). 18160987 10.1371/journal.pntd.0000088 PMC2154385

[37] Klion AD, Massougbodji A, Sadeler BC, Ottesen EA, Nutman TB. Loiasis in endemic and nonendemic populations: immunologically mediated differences in clinical presentation. J Infect Dis. 1991;163(6):1318–25. 10.1093/infdis/163.6.1318. (PubMed PMID: 2037798). 2037798 10.1093/infdis/163.6.1318

[38] Sharp D. Loa loa infections. A case with rapid onset of symptoms. Lancet. 1929;214:765–6.

[39] Thomas J, Chastel C, Forcain L. [Clinical and parasitic latency in filariasis due to Loa loa and Onchocerca Volvulus]. Bull Soc Pathol Exot Filiales. 1970;63(1):90–4. (PubMed PMID PMID: 5468323). 5468323

[40] Veletzky L, Eberhardt KA, Hergeth J, Stelzl DR, Zoleko Manego R, Mombo-Ngoma G, et al. Distinct loiasis infection states and associated clinical and hematological manifestations in patients from Gabon. PLoS Negl Trop Dis. 2022;16(9):e0010793. 10.1371/journal.pntd.0010793. (PubMed PMID: 36121900; PubMed Central PMCID: PMC9521832). 36121900 10.1371/journal.pntd.0010793 PMC9521832

[41] Eveland LK, Yermakov V, Kenney M. Loa loa infection without microfilaraemia. Trans R Soc Trop Med Hyg. 1975;69(3):354–5. 10.1016/0035-9203(75)90131-5. (PubMed PMID: 1058558). 1058558 10.1016/0035-9203(75)90131-5

[42] Richardson ET, Luo R, Fink DL, Nutman TB, Geisse JK, Barry M. Transient facial swellings in a patient with a remote African travel history. J Travel Med. 2012;19(3):183–5. 10.1111/j.1708-8305.2012.00612.x. (PubMed PMID: 22530826; PubMed Central PMCID: PMC3437052). 22530826 10.1111/j.1708-8305.2012.00612.x PMC3437052

[43] Bouyou Akotet MK, Owono-Medang M, Mawili-Mboumba DP, Moussavou-Boussougou MN, Nzenze Afène S, Kendjo E, et al. The relationship between microfilaraemic and amicrofilaraemic loiasis involving co-infection with Mansonella perstans and clinical symptoms in an exposed population from Gabon. J Helminthol. 2016;90(4):469–75. 10.1017/s0022149x15000607. (Epub 20150813. PubMed PMID: 26268068). 26268068 10.1017/S0022149X15000607

[44] Toure FS, Deloron P, Egwang TG, Wahl G. [Relationship between the intensity of Loa loa filariasis transmission and prevalence of infections]. Med Trop (Mars). 1999;59(3):249–52. (PubMed PMID: 10701202). 10701202

[45] Garcia A, Abel L, Cot M, Richard P, Ranque S, Feingold J, et al. Genetic epidemiology of host predisposition microfilaraemia in human loiasis. Trop Med Int Health. 1999;4(8):565–74. 10.1046/j.1365-3156.1999.00442..x. (PubMed PMID: 10499080). 10499080 10.1046/j.1365-3156.1999.00442.x

[46] Eyebe S, Sabbagh A, Pion SD, Nana-Djeunga HC, Kamgno J, Boussinesq M, et al. Familial aggregation and heritability of Loa loa Microfilaremia. Clin Infect Dis. 2018;66(5):751–7. 10.1093/cid/cix877. (PubMed PMID: 29040446). 29040446 10.1093/cid/cix877

[47] Klion AD. Filarial infections in travelers and immigrants. Curr Infect Dis Rep. 2008;10(1):50–7. 10.1007/s11908-008-0010-2. (PubMed PMID: 18377816). 10.1007/s11908-008-0010-2 18377816

[48] Pinder M. Loa loa - a neglected filaria. Parasitol Today. 1988;4(10):279–84. 10.1016/0169-4758(88)90019-1.). (PubMed PMID: 15463001. 15463001 10.1016/0169-4758(88)90019-1

[49] Fain A. [Current problems of loaiasis]. Bull World Health Organ. 1978;56(2):155–67. (PubMed PMID: 96952; PubMed Central PMCID: PMC2395560). 96952 PMC2395560

[50] Hildebrandt TR, Ramharter H, Kabwende AL, Endamne LR, Davi SD, Adegnika AA, et al. Recurring transient tooth pain as newly described symptom of migratory loiasis: a mixed-methods study in rural Gabon. Am J Trop Med Hyg. 2024. 10.4269/ajtmh.24-0059. (PubMed PMID: 39043172). 39043172 10.4269/ajtmh.24-0059 PMC11448542

[51] Akue JP. Encephalitis due to Loa loa. In: Tkachev S, editor. Non-Flavivirus Encephalitis. 2011. pp. 341– 60.

[52] Lukiana T, Mandina M, Situakibanza NH, Mbula MM, Lepira BF, Odio WT, et al. A possible case of spontaneous Loa loa encephalopathy associated with a glomerulopathy. Filaria J. 2006;5:6. 10.1186/1475-2883-5-6. (PubMed PMID: 16686951; PubMed Central PMCID: PMC1471781). 16686951 10.1186/1475-2883-5-6 PMC1471781

[53] Arrey-Agbor DB, Nana-Djeunga HC, Mogoung-Wafo AE, Mafo M, Danwe C, Kamgno J. Case Report: probable case of spontaneous Encephalopathy due to Loiasis and dramatic reduction of Loa loa Microfilariaemia with prolonged repeated courses of Albendazole. Am J Trop Med Hyg. 2018;99(1):112–5. 10.4269/ajtmh.17-0664. (PubMed PMID: 29741149; PubMed Central PMCID: PMC6085801). 29741149 10.4269/ajtmh.17-0664 PMC6085801

[54] Van Bogaert L, Dubois A, Janssens PG, Radermecker J, Tverdy G, Wanson M. Encephalitis in loa-loa filariasis. J Neurol Neurosurg Psychiatry. 1955;18(2):103–19. 10.1136/jnnp.18.2.103. (PubMed PMID: 14381919; PubMed Central PMCID: PMC503223). 14381919 10.1136/jnnp.18.2.103 PMC503223

[55] Zuidema PJ. Renal changes in loiasis. Folia Med Neerl. 1971;14(4):168–72. 4941768

[56] Katner H, Beyt BE Jr., Krotoski WA. Loiasis and renal failure. South Med J. 1984;77(7):907–8. 10.1097/00007611-198407000-00027. (PubMed PMID: 6588571). 6588571 10.1097/00007611-198407000-00027

[57] Buell KG, Whittaker C, Chesnais CB, Jewell PD, Pion SDS, Walker M, et al. Atypical clinical manifestations of Loiasis and their relevance for endemic populations. Open Forum Infect Dis. 2019;6(11):ofz417. 10.1093/ofid/ofz417. (PubMed PMID: 31696139; PubMed Central PMCID: PMC6824532). 31696139 10.1093/ofid/ofz417 PMC6824532

[58] Tamarozzi F, Buonfrate D, Ricaboni D, Ursini T, Foti G, Gobbi F. Spleen nodules in Loa loa infection: re-emerging knowledge and future perspectives. Lancet Infect Dis. 2022;22(7):e197–206. 10.1016/s1473-3099(21)00632-0. (Epub 20220224. PMID: 35219405). 35219405 10.1016/S1473-3099(21)00632-0

[59] Mombo-Ngoma G, Mackanga JR, Basra A, Capan M, Manego RZ, Adegnika AA, et al. Loa loa infection in pregnant women, Gabon. Emerg Infect Dis. 2015;21(5):a899–901. 10.3201/eid2105.141471. (PubMed PMID: 25897819; PubMed Central PMCID: PMC4412224). 10.3201/eid2105.141471 PMC4412224 25897819

[60] Otranto D, Eberhard ML. Zoonotic helminths affecting the human eye. Parasit Vectors. 2011;4:41. 10.1186/1756-3305-4-41. (PubMed PMID: 21429191; PubMed Central PMCID: PMC3071329). 21429191 10.1186/1756-3305-4-41 PMC3071329

[61] Campillo JT, Louya F, Bikita P, Missamou F, Pion SDS, Boussinesq M, et al. Factors associated with the periodicity of Loa loa microfilaremia in the Republic of the Congo. Parasit Vectors. 2022;15(1):417. 10.1186/s13071-022-05541-y. (PubMed PMID: 36352480; PubMed Central PMCID: PMC9647901). 36352480 10.1186/s13071-022-05541-y PMC9647901

[62] Mischlinger J, Manego RZ, Mombo-Ngoma G, Ekoka Mbassi D, Hackbarth N, Ekoka Mbassi FA, et al. Diagnostic performance of capillary and venous blood samples in the detection of Loa loa and Mansonella perstans microfilaraemia using light microscopy. PLoS Negl Trop Dis. 2021;15(8):e0009623. 10.1371/journal.pntd.0009623. (PubMed PMID: 34398886; PubMed Central PMCID: PMC8389422). 34398886 10.1371/journal.pntd.0009623 PMC8389422

[63] Mbassi FE, Mombo-Ngoma G, Ndoumba WN, Yovo EK, Eberhardt KA, Mbassi DE, et al. Performance of Field’s Stain compared with conventional Giemsa Stain for the Rapid Detection of Blood Microfilariae in Gabon. Am J Trop Med Hyg. 2022;107(2):383–7. 10.4269/ajtmh.22-0061. (PubMed PMID: 35895407; PubMed Central PMCID: PMC9393457). 35895407 10.4269/ajtmh.22-0061 PMC9393457

[64] Mathison BA, Couturier MR, Pritt BS. Diagnostic identification and differentiation of Microfilariae. J Clin Microbiol. 2019;57(10):101128. 10.1128/jcm.00706-19. (PubMed PMID: PMID: 31340993; PubMed Central PMCID: PMC6760958). /jcm.00706– 19. 10.1128/JCM.00706-19 PMC6760958 31340993

[65] Formenti F, Tang TT, Tamarozzi F, Silva R, La Marca G, Pajola B, et al. Preliminary comparison between an in-house real-time PCR vs microscopy for the diagnosis of Loa loa and Mansonella perstans. Acta Trop. 2021;216:105838. 10.1016/j.actatropica.2021.105838. (Epub 20210121. PubMed PMID: 33484727). 33484727 10.1016/j.actatropica.2021.105838

[66] Ta-Tang TH, Febrer-Sendra B, Berzosa P, Rubio JM, Romay-Barja M, Ncogo P, et al. Comparison of three PCR-based methods to detect Loa loa and Mansonella perstans in long-term frozen storage dried blood spots. Trop Med Int Health. 2022;27(8):686–95. 10.1111/tmi.13786. (Epub 20220621.PMID: 35653502). 35653502 10.1111/tmi.13786

[67] Touré FS, Mavoungou E, Deloron P, Egwang TG. [Comparative analysis of 2 diagnostic methods of human loiasis: IgG4 serology and nested PCR]. Bull Soc Pathol Exot. 1999;92(3):167–70. (PubMed PMID: 10472442). 10472442

[68] Kamgno J, Pion SD, Chesnais CB, Bakalar MH, D’Ambrosio MV, Mackenzie CD, et al. A test-and-not-treat strategy for Onchocerciasis in Loa loa-endemic areas. N Engl J Med. 2017;377(21):2044–52. 10.1056/NEJMoa1705026. (PubMed PMID: 29116890; PubMed Central PMCID: PMC5629452). 29116890 10.1056/NEJMoa1705026 PMC5629452

[69] Veletzky L, Eberhardt KA, Hergeth J, Stelzl DR, Zoleko Manego R, Kreuzmair R, et al. Analysis of diagnostic test outcomes in a large loiasis cohort from an endemic region: serological tests are often false negative in hyper-microfilaremic infections. PLoS Negl Trop Dis. 2024;18(3):e0012054. 10.1371/journal.pntd.0012054. (PubMed PMCID: PubMed Central PMC10965051). 38484012 10.1371/journal.pntd.0012054 PMC10965051

[70] Dieki R, Eyang Assengone ER, Nsi Emvo E, Akue JP. Profile of loiasis infection through clinical and laboratory diagnostics: the importance of biomarkers. Trans R Soc Trop Med Hyg. 2023;117(5):349–. 10.1093/trstmh/trac116. (PubMed PMID: 36520072; PubMed Central PMCID: PMC10153730). 57. 36520072 10.1093/trstmh/trac116 PMC10153730

[71] Pedram B, Pasquetto V, Drame PM, Ji Y, Gonzalez-Moa MJ, Baldwin RK, et al. A novel rapid test for detecting antibody responses to Loa loa infections. PLoS Negl Trop Dis. 2017;11(7):e0005741. 10.1371/journal.pntd.0005741. (PubMed PMID: 28749939; PubMed Central PMCID: PMC5531435). 28749939 10.1371/journal.pntd.0005741 PMC5531435

[72] Zoleko-Manego R, Kreuzmair R, Veletzky L, Ndzebe-Ndoumba W, Ekoka Mbassi D, Okwu DG, et al. Efficacy, safety, and tolerability of albendazole and ivermectin based regimens for the treatment of microfilaraemic loiasis in adult patients in Gabon: a randomized controlled assessor blinded clinical trial. PLoS Negl Trop Dis. 2023;17(8):e0011584. 10.1371/journal.pntd.0011584. (PubMed PMID: 37639396; PubMed Central PMCID: PMC10491396). 37639396 10.1371/journal.pntd.0011584 PMC10491396

[73] Hawking F. Diethylcarbamazine and new compounds for the treatment of filariasis. Adv Pharmacol Chemother. 1979;16:130–94. (PubMed PMID: 382798). 10.1016/s1054-3589(08)60244-6 382798

[74] Mastrandrea G, Sanguigni S. [The Mazzotti test with diethylcarbamazine in some cases of filariasis]. Arch Ital Sci Med Trop Parassitol. 1968;49(7):195–202. (PubMed PMID: 5715933. 5715933

[75] Chai JY, Jung BK, Hong SJ. Albendazole and Mebendazole as anti-parasitic and anti-cancer agents: an update. Korean J Parasitol. 2021;59(3):189–225. 10.3347/kjp.2021.59.3.189. (Epub 20210621. PubMed PMID: 34218593; PubMed Central PMCID: PMC8255490). 34218593 10.3347/kjp.2021.59.3.189 PMC8255490

[76] Chesnais CB, Pion SD, Boulle C, Gardon J, Gardon-Wendel N, Fokom-Domgue J, et al. Individual risk of post-ivermectin serious adverse events in subjects infected with Loa loa. EClinicalMedicine. 2020;28:100582. 10.1016/j.eclinm.2020.100582. (PubMed PMID: 33294807; PubMed Central PMCID: PMC7700892). 33294807 10.1016/j.eclinm.2020.100582 PMC7700892

[77] Gardon J, Gardon-Wendel N, Demanga N, Kamgno J, Chippaux JP, Boussinesq M. Serious reactions after mass treatment of onchocerciasis with ivermectin in an area endemic for Loa loa infection. Lancet. 1997;350(9070):18–22. 10.1016/s0140-6736(96)11094-1. (PubMed PMID: 9217715). 9217715 10.1016/S0140-6736(96)11094-1

[78] Herrick JA, Legrand F, Gounoue R, Nchinda G, Montavon C, Bopda J, et al. Posttreatment reactions after single-dose diethylcarbamazine or ivermectin in subjects with Loa loa infection. Clin Infect Dis. 2017;64(8):1017–25. 10.1093/cid/cix016. (PubMed PMID: 28329346; PubMed Central PMCID: PMC5850646). 28329346 10.1093/cid/cix016 PMC5850646

[79] Klion AD, Ottesen EA, Nutman TB. Effectiveness of diethylcarbamazine in treating loiasis acquired by expatriate visitors to endemic regions: long-term follow-up. J Infect Dis. 1994;169(3):604–10. 10.1093/infdis/169.3.604. (PubMed PMID: 8158033). 8158033 10.1093/infdis/169.3.604

[80] Klion AD, Horton J, Nutman TB. Albendazole therapy for loiasis refractory to diethylcarbamazine treatment. Clin Infect Dis. 1999;29(3):680–2. 10.1086/598654. (PubMed PMID: 10530467). 10530467 10.1086/598654

[81] Gobbi F, Bottieau E, Bouchaud O, Buonfrate D, Salvador F, Rojo-Marcos G, et al. Comparison of different drug regimens for the treatment of loiasis-A TropNet retrospective study. PLoS Negl Trop Dis. 2018;12(11):e0006917. 10.1371/journal.pntd.0006917. (PubMed PMID: 30383753; PubMed Central PMCID: PMC6233929). 30383753 10.1371/journal.pntd.0006917 PMC6233929

[82] Klion AD, Massougbodji A, Horton J, Ekoue S, Lanmasso T, Ahouissou NL, et al. Albendazole in human loiasis: results of a double-blind, placebo-controlled trial. J Infect Dis. 1993;168(1):202–6. (PubMed PMID: 8515109). 8515109 10.1093/infdis/168.1.202

[83] Gobbi F, Buonfrate D, Tamarozzi F, Degani M, Angheben A, Bisoffi Z. Efficacy of high-dose albendazole with Ivermectin for Treating Imported Loiasis, Italy. Emerg Infect Dis. 2019;25(8):1574–6. 10.3201/eid2508.190011. (PubMed PMID: 31310225; PubMed Central PMCID: PMC6649345). 31310225 10.3201/eid2508.190011 PMC6649345

[84] Grobusch MP, Kombila M, Autenrieth I, Mehlhorn H, Kremsner PG. No evidence of Wolbachia endosymbiosis with Loa loa and Mansonella perstans. Parasitol Res. 2003;90(5):405–8. 10.1007/s00436-003-0872-z. (Epub 20030514. PubMed PMID: 12748849). 12748849 10.1007/s00436-003-0872-z

[85] Büttner DW, Wanji S, Bazzocchi C, Bain O, Fischer P. Obligatory symbiotic Wolbachia endobacteria are absent from Loa loa. Filaria J.2003;2(1):10. 10.1186/1475-2883-2-10. (PubMed PMID: 12801420; PubM;ed Central PMCID: PMC161789) 10.1186/1475-2883-2-10 PMC161789 12801420

[86] O'Connell EM, Nutman TB. Reduction of Loa loa Microfilaremia with Imatinib - A Case Report. N Engl J Med. 2017;377(21):2095-6. 10.1056/NEJMc1712990. (PubMed PMID: 29166233; PubMed Central PMCID: PMC5774668) 10.1056/NEJMc1712990 PMC5774668 29166233

[87] National Library of Medicine. Efficacy and Microfilaricidal Kinetics of Imatinib for the Treatment of Loa loa. https://clinicaltrials.gov/ct2/show/record/NCT026445252015. Accessed 25 Sep 2024.

[88] Wafeu GS, Lepage TM, Campillo JT, Efon-Ekangouo A, Nana-Djeunga H-C, Nzune-Toche N, et al. Safety and short-term efficacy of a single dose of 2 mg moxidectin in Loa loa infected individuals: a double-blind, randomized ivermectin-controlled trial with ascending microfilarial densities. Open Forum Infect Dis. 2024. 10.1093/ofid/ofae240. (PubMed PMID: 38966851; PubMed Central PMCID: PMC11222972). 38966851 10.1093/ofid/ofae240 PMC11222972

[89] Okonkwo ON, Hassan AO, Alarape T, Akanbi T, Oderinlo O, Akinye A, et al. Removal of adult subconjunctival Loa loa amongst urban dwellers in Nigeria. PLoS Negl Trop Dis. 2018;12(11):e0006920. 10.1371/journal.pntd.0006920. (PubMed PMID: 30427837; PubMed Central PMCID: PMC6261630). 30427837 10.1371/journal.pntd.0006920 PMC6261630

[90] Odedra A, Lalloo DG, Kennedy G, Llewellyn S, McCarthy JS. Safety and effectiveness of apheresis in the treatment of infectious diseases: a systematic review. J Infect. 2019;79(6):513–20. 10.1016/j.jinf.2019.09.014. (Epub 20191014. PMID: 31622632). 31622632 10.1016/j.jinf.2019.09.014

[91] Legrand F, Herrick J, Makiya M, Ramanathan R, Thompson R, Rampertaap S, et al. A Randomized, Placebo-controlled, double-blind pilot study of single-dose humanized Anti-IL5 antibody (Reslizumab) for the reduction of Eosinophilia following Diethylcarbamazine Treatment of Loa loa infection. Clin Infect Dis. 2021;73(7):e1624–31. 10.1093/cid/ciaa1365. (PubMed PMID: 32910141; PubMed Central PMCID: PMC8677597). 32910141 10.1093/cid/ciaa1365 PMC8677597

[92] Andy JJ, Bishara FF, Soyinka OO, Odesanmi WO. Loasis as a possible trigger of African endomyocardial fibrosis: a case report from Nigeria. Acta Trop. 1981;38(2):179–86. (PubMed PMID: 6115557). 6115557

[93] Ive FA, Willis AJ, Ikeme AC, Brockington IF. Endomyocardial fibrosis and filariasis. Q J Med. 1967;36(144):495–516. (PubMed PMID: 6077228). 6077228

[94] Duke BO. Studies on the chemoprophylaxis of loiasis. I. Experiments on monkeys, with special reference to diethylcarbamazine (Banocide). Ann Trop Med Parasitol. 1961;55. 10.1080/00034983.1961.11686073. (PubMed PMID: 13888307).:447– 51. 10.1080/00034983.1961.11686073 13888307

[95] Nutman TB, Miller KD, Mulligan M, Reinhardt GN, Currie BJ, Steel C, et al. Diethylcarbamazine prophylaxis for human loiasis. Results of a double-blind study. N Engl J Med. 1988;319(12):752–6. 10.1056/nejm198809223191204. (PubMed PMID: 3166107). 3166107 10.1056/NEJM198809223191204

[96] Drews SJ, Spencer BR, Wendel S, Bloch EM. Filariasis and transfusion-associated risk: a literature review. Vox Sang. 2021;116(7):741–54. 10.1111/vox.13073. (Epub 20210125. PubMed PMID: 33491765). 33491765 10.1111/vox.13073

